# Reciprocal regulation of MicroRNA-99a and insulin-like growth factor I receptor signaling in oral squamous cell carcinoma cells

**DOI:** 10.1186/1476-4598-13-6

**Published:** 2014-01-10

**Authors:** Yi-Chen Yen, Shine-Gwo Shiah, Hsiao-Chien Chu, Yuan-Ming Hsu, Jenn-Ren Hsiao, Jang-Yang Chang, Wen-Chun Hung, Chun-Ta Liao, Ann-Joy Cheng, Ya-Ching Lu, Ya-Wen Chen

**Affiliations:** 1 National Institute of Cancer Research, National Health Research Institutes, Miaoli, Taiwan; 2 Department of Otolaryngology, National Cheng Kung University Hospital, College of Medicine, National Cheng Kung University, Tainan, Taiwan; 3 National Institute of Cancer Research, National Health Research Institutes, Tainan, Taiwan; 4 Department of Internal Medicine, National Cheng Kung University Hospital, College of Medicine, Tainan, Taiwan; 5 Department of Otorhinolaryngology, Head and Neck Surgery, Chang Gung Memorial Hospital and Chang Gung University, Taoyuan, Taiwan; 6 Department of Medical Biotechnology, Chang Gung University, Taoyuan, Taiwan; 7 Graduate Institute of Basic Medical Science, China Medical University, Taichung, Taiwan

**Keywords:** MicroRNA-99a, Tumor metastasis suppressor, Insulin-like growth factor 1 receptor, Reciprocal regulation, Oral squamous cell carcinoma

## Abstract

**Background:**

MicroRNAs (miRNAs), small noncoding RNA molecules can function as oncogenes or tumor suppressors in tumorigenesis. Oral squamous cell carcinoma (OSCC) is one of the most prevalent cancers worldwide with a 5-year survival rate of approximately 50%.

**Methods:**

The expression of microRNA-99a (miR-99a) in OSCC tissues and cell lines was investigated using quantitative reverse transcription-polymerase chain reaction (qRT-PCR) analysis. The functions of miR-99a in migration/invasion and lung colonization were determined by transwell and tail vein injection assays, respectively. Specific targets of miR-99a were determined by software prediction, correlation with target protein expression, and luciferase reporter assay. The signaling pathways involved in regulation of miR-99a were investigated using the kinase inhibitors.

**Results:**

We observed reduced levels of miR-99a, identified as one of the most downregulated miRNA in OSCC and all tested OSCC cell lines compared to normal oral keratinocytes. Ectopic miR-99a expression in OSCC cells markedly reduced migration and invasion in vitro as well as lung colonization in vivo. When evaluating the specific targets of miR-99a, we found that ectopic miR-99a expression downregulates insulin-like growth factor 1 receptor (IGF1R) protein and that the expression of miR-99a correlates negatively with IGF1R protein in OSCC cells. Insertion of the 3′UTR of IGF1R mRNA into the 3′UTR of a reporter gene markedly reduced luciferase activity in OSCC cells expressing miR-99a, suggesting that miR-99a reduces luciferase activity by targeting the 3′UTR of IGF1R mRNA. When evaluating the mechanisms of miR-99a downregulation, we observed the upregulation of miR-99a expression in serum-starved conditions and its suppression in response to insulin-like growth factor (IGF1) stimulation. Inhibitors of phosphatidylinositol 3-kinase (PI3K) and mitogen-activated protein kinase (MAPK) kinase inhibited IGF1-induced suppression of miR-99a, suggesting the negative regulation of miR-99a expression by IGF1R signaling.

**Conclusion:**

Overall, results indicate that miR-99a functions as a tumor metastasis suppressor in OSCC cells and mutually regulates IGF1R expression in a reciprocal regulation.

## Introduction

Oral cancer is one of the most common cancers worldwide with an annual estimated incidence of approximately 275 000. More than 90% of oral cancers are squamous cell carcinomas arising in the oral cavity [1]. The initiation and progression of OSCC involve a multistep process of aberrant genetic events following the action of various carcinogens, which might be derived from the chronic use of tobacco, alcohol, and betel quid [2]. Cigarette smoking and alcohol drinking are the major risk factors for OSCC in western countries. In southeast Asia, betel quid chewing and cigarette smoking are the predominant risk factors for OSCC [2]. Currently, the treatment of OSCC is problematic. In most countries, the 5-year survival rate for oral cancer is approximately 50% [1], with locoregional recurrence and/or distant metastasis the major causes of death in these patients [3]. To improve the treatment outcome, elucidation of the molecular mechanisms involved in the carcinogenesis and progression of OSCC is, therefore, needed.

In recent years, biomedical investigation has increasingly focused on a relatively small number of microRNAs (miRNAs), noncoding RNAs approximately 17 to 25 nucleotides in length [4]. These miRNAs are transcribed by RNA polymerase II in hairpin structures and processed by RNase III Drosha into long precursor miRNAs (pre-miRNAs) in the nucleus [4]. The precursors are then transported into the cytoplasm and processed by RNase III Dicer to form mature miRNA, which regulates gene expression by controlling mRNA translation and stability. It does this through the translational repression of its target mRNA, or by increasing its degradation using an RNA interference mechanism [4]. Evidence suggests that miRNAs can be functionally classified as proto-oncogenes or tumor suppressor genes and aberrantly expressed in different cancer types Dysregulation of these cancerous miRNAs is involved in tumor initiation and progression by facilitating an inappropriate cellular program that promotes uncontrolled cell proliferation, favors survival, inhibits differentiation, or induces invasive behavior [5-7].

Previous studies characterized microRNA-99a (miR-99a) as a tumor suppressor in several human cancers, including childhood adrenocortical tumors [8], prostate [9], liver [10], head and neck [11], and oral [12] cancers. These studies showed that expression of miR-99a could reduce the expression of a number of proto-oncogenes by targeting their 3′ untranslated region (3′UTR) [8-13]. Several groups further identified the downregulation of miR-99a in clinical samples of oral or head and neck squamous cell carcinoma (HNSCC) of different stages using miRNA microarray analysis [11,12,14-16]. However, most of the biological functions of miR-99a in OSCC remain unknown.

Considering the diverse and overlapping biological functions of the identified targets of miR-99a, it is possible that an additional miR-99a target might contribute to OSCC tumorigenesis. In this study, we identified IGF1R as a specific target of miR-99a. The IGF1R is overexpressed in several cancers, including in OSCC tissues and cell lines [17,18], and essential for malignant transformation and progression [19]. In the study by Lara et al., IGF1R expression was a predictor of clinical outcome in patients with locally advanced OSCC [20].

Insulin-like growth factor 1 receptor is a transmembrane tyrosine kinase receptor with a heterodimer of α- and β-chains, and activated by its ligands insulin–like growth factor 1 (IGF1) and insulin-like growth factor 2 (IGF2) [21]. Insulin-like growth factor 1 receptor signaling pathways influence cancer cell proliferation, adhesion, migration, and cell death, and are critical in tumor cell survival and metastasis [22]. Activation of IGF1R leads to activation of the Ras, Raf, and mitogen-activated protein kinase (MAPK) pathway, resulting in increased proliferation and stimulation of the phosphatidylinositol 3-kinase (PI3K) pathway, which subsequently leads to the inhibition of apoptosis [23]. The overexpression of a constitutively active form of IGF1R in the mouse mammary gland can initiate tumorigenesis [24], whereas expression of a dominant negative IGF1R inhibits Ras-induced cell transformation [25]. In this study, we demonstrated that miR-99a is downregulated in OSCC tissues and cell lines and can function as a tumor metastasis suppressor for migration, invasion and lung colonization in OSCC cells. Using bioinformatic prediction and luciferase reporter assays, we further confirmed that miR-99a negatively regulates IGF1R protein levels by specifically binding to the 3′UTR of IGF1R mRNA. We identified the negative regulation of miR-99a expression by IGF1-induced signaling and proposed a reciprocal regulation responsible for the mutual regulation of miR-99a and IGF1R.

## Results

### Downregulation of miR-99a in OSCC patients and cell lines

**Figure 1:**
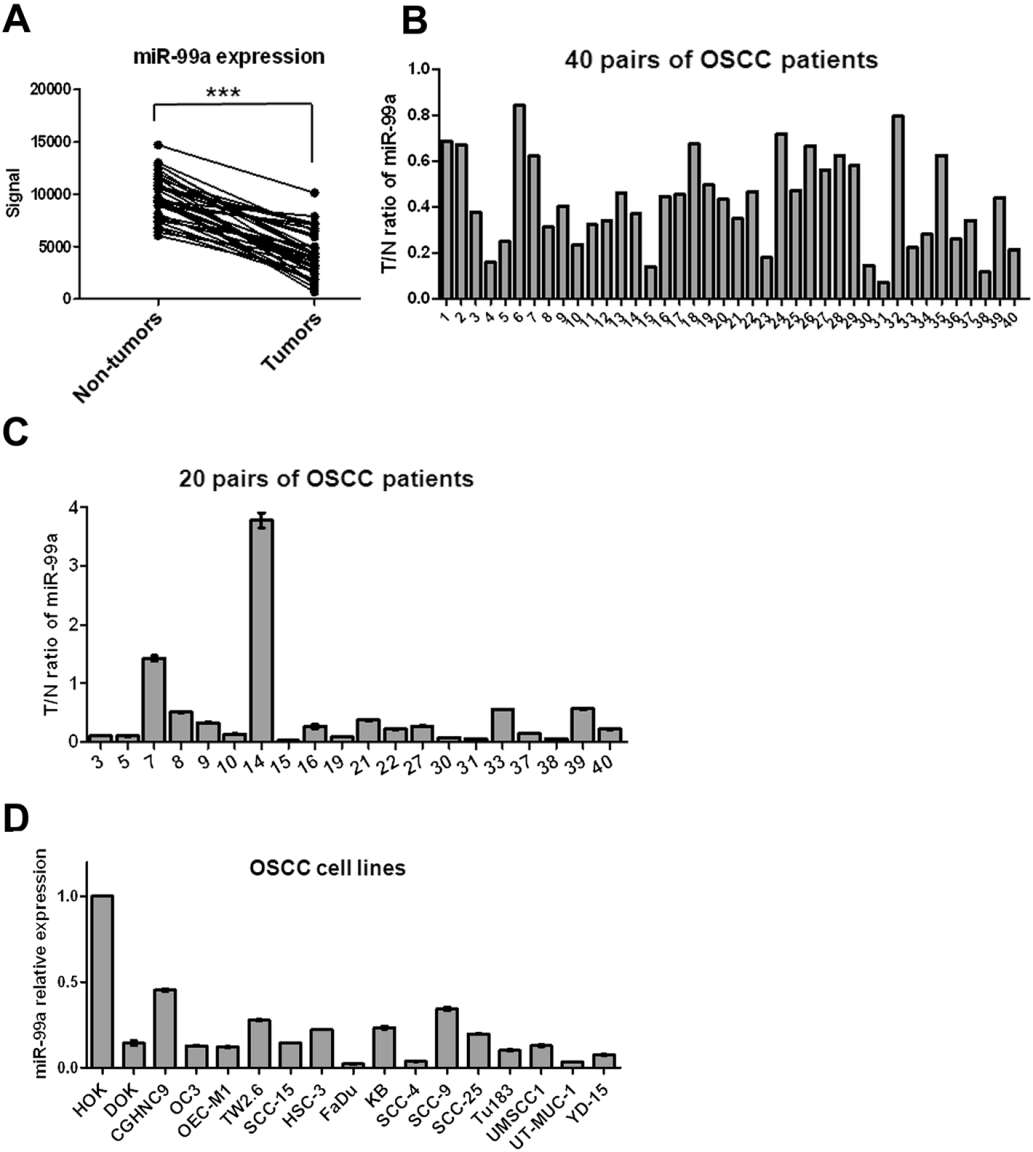
**Downregulation of miR-99a in OSCC tissues and cell lines. (A)** MiR-99a was downregulated significantly in OSCC tissues compared to nontumorous tissues. *** p < 0.001. **(B)** Microarray analysis revealed the significant downregulation of miR-99a expression in 28/40 (70%) OSCC tissues, with a >2-fold decrease in expression in comparison with the corresponding nontumorous tissues. The relative miR-99a expression was determined by dividing the detected signal from a tumorous tissue by that from its corresponding nontumorous tissue. **(C)** Validation of miR-99a expression in 20 pairs of OSCC tissues using qRT-PCR analysis. Expression of miR-99a was normalized against an endogenous control U6. The relative expression of miR-99a was determined by normalizing the expression of miR-99a in a tumorous tissue to that in its corresponding nontumorous tissue. Bar, SE. **(D)** The relative expression of miR-99a in 16 OSCC cell lines and one HOK cell line was evaluated using qRT-PCR analysis. Expression of miR-99a was normalized against an endogenous control U6. The relative expression of miR-99a was determined by normalizing the expression of miR-99a in OSCC cell lines to that in HOK. Bar, SE.

We examined the miRNA expression profiles of 40 pairs of OSCC tissue specimens and their corresponding nontumorous epithelia, with results identifying miR-99a as one of the most downregulated miRNAs (GSE45238). The expression of miR-99a was reduced significantly in OSCC samples in comparison with the adjacent noncancerous tissues (Figure 1A). Twenty-eight of 40 (70%) OSCC tissue samples exhibited a >2-fold decrease in miR-99a expression relative to their corresponding nontumorous tissues (Figure 1B). These findings were confirmed by results from quantitative reverse-transcription polymerase chain reaction (qRT-PCR) analysis using 20 pairs of OSCC samples and their corresponding nontumorous tissues. Consistent with these data, we detected reduced levels of miR-99a in most of the tested samples and 15/20 (75%) OSCC tissues displayed a >2-fold decrease in miR-99a expression compared to their corresponding nontumorous tissues (Figure 1C). We also used qRT-PCR to examine the expression of miR-99a in 16 OSCC cell lines, observing reduced levels of miR-99a in all OSCC cell lines when compared with normal human oral keratinocytes (HOK) (Figure 1D). To address the clinical significance of downregulated miR-99a in OSCC, we investigated the correlation between the clinicopathological features of OSCC and miR-99a expression using 2-tailed student’s *t* test. We observed insignificant correlation between clinicopathological parameters, including pathological stage, and tumor status (Additional file 1: Table S1). Interestingly, the levels of miR-99a expression displayed significantly lower in OSCC with lymphovascular invasion than in OSCC without lymphovascular invasion (p = 0.0144) (Additional file 1: Table S1), suggesting a role of miR-99a in lymphovascular invasion. The identification of significant reductions in miR-99a expression in OSCC tissues and cell lines compared to nontumorous tissues and HOK cells suggested that miR-99a has possible pathological roles in OSCC.

### MiR-99a inhibits migration, invasion and lung colonization in OSCC cells

**Figure 2:**
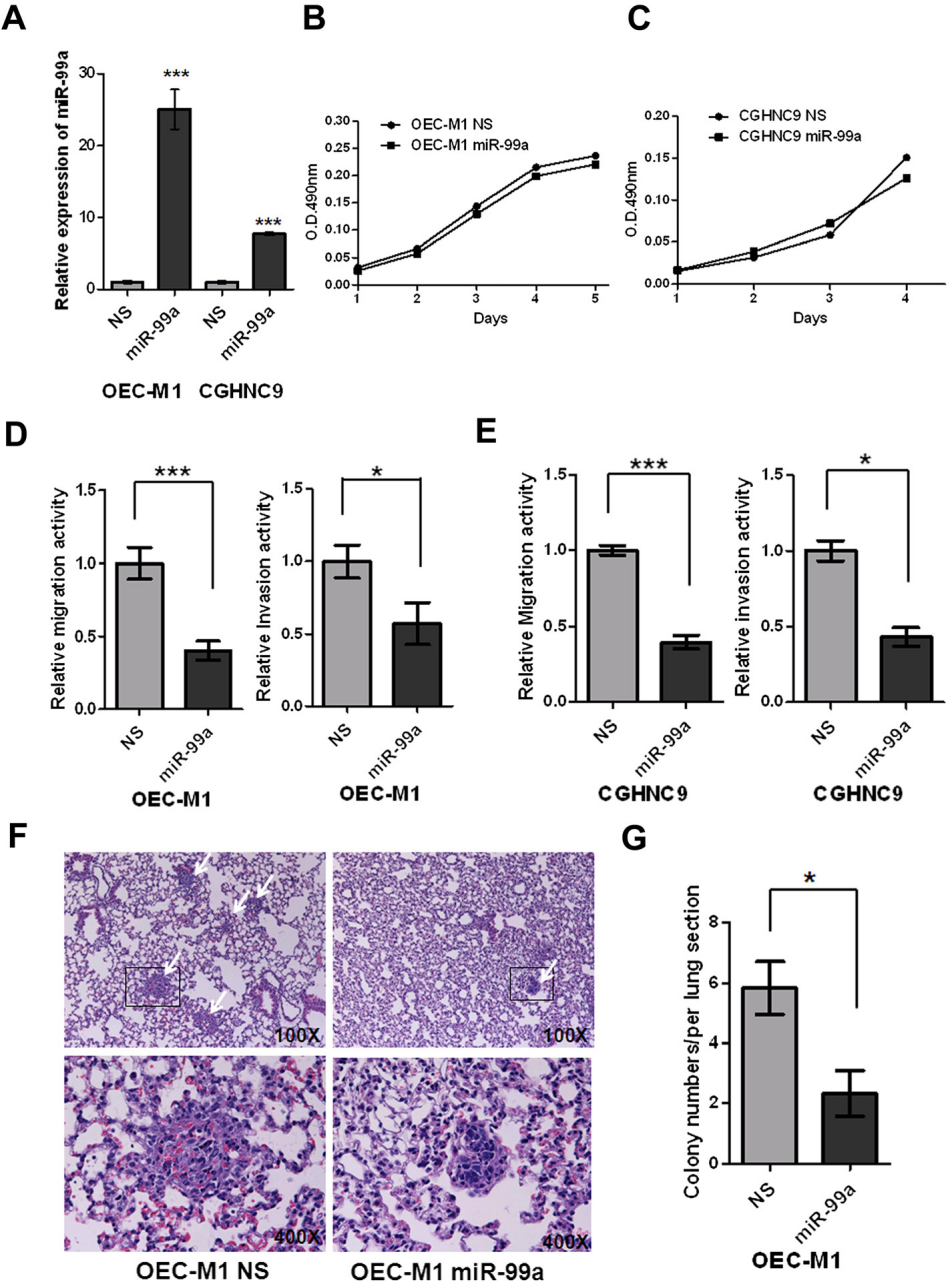
**Ectopic miR-99a expression suppressed migration, invasion and lung colonization. (A)** Levels of miR-99a in OEC-M1 and CGHNC9 cells with ectopic miR-99a expression (OEC-M1 miR-99a and CGHNC9 miR-99a) and their corresponding controls with lentiviral non-silencing microRNA (OEC-M1 NS and CGHNC9 NS) were determined using qRT-PCR analysis. MiR-99a expression was normalized against an endogenous control U6. The relative expression of miR-99a was determined by normalizing the expression of miR-99a in cells with ectopic miR-99a expression (miR-99a) to that in control cells (NS). Bar, SE; ***p < 0.001. **(B)** Cell proliferation was analyzed in OEC-M1 cells with ectopic miR-99a expression (OEC-M1 miR-99a) and controls (OEC-M1 NS). Bar, SE. **(C)** Cell proliferation was evaluated in CGHNC9 cells with ectopic miR-99a expression (CGHNC9 miR-99a) and controls (CGHNC9 NS). Bar, SE. **(D)** The relative migration/invasion activity of OEC-M1 cells expressing miR-99a (OEC-M1 miR-99a) and controls (OEC-M1 NS). Bar, SE; * p < 0.05; ***p < 0.001. **(E)** The relative migration/invasion activity of CGHNC9 cells expressing miR-99a (CGHNC9 miR-99a) and controls (CGHNC9 NS). Bar, SE; * p < 0.05; ***p < 0.001. **(F)** Representative lung field of nude mice after delivery via tail vein injection of OEC-M1 cells with ectopic miR-99a expression (OEC-M1 miR-99a) or controls (OEC-M1 NS). The white arrowhead indicates tumor nodule in the lung fields. The boxed area in the upper panel is shown at higher magnification in the lower panel. **(G)** Quantification of colony number/per lung section in mice injected with miR-99a expressing OEC-M1 (OEC-M1 miR-99a) or controls (OEC-M1 NS). Bar, SE; *p < 0.05.

To further investigate the biological functions of miR-99a, we overexpressed miR-99a in OSCC cell lines using lentiviral infection, and then analyzed the cells using qRT-PCR. Results indicated that ectopic miR-99a expression in OEC-M1 and CGHNC9 cells, established from Taiwan OSCC patients, led to increased miR-99a expression (Figure 2A) and insignificant reductions in cell growth (Figures 2B and 2C) when compared with their corresponding controls. Using transwell assay, we then identified that migration and invasion activities were reduced significantly in the OEC-M1 cells with ectopic miR-99a expression when compared with their corresponding controls (Figure 2D). We observed similar results in CGHNC9 cells with ectopic miR-99a expression (Figure 2E). To determine whether the effects of miR-99a on migration and invasion correlated with lung colonization, we injected OSCC cells into mice via tail vein injection. We observed that ectopic miR-99a expression led to decreased colony number of tumor nodules in the lungs (2.33 ± 0.76 versus 5.83 ± 0.87/per lung section for vector control) (Figures 2F and 2G). These data indicated that miR-99a functions as a tumor metastasis suppressor of migration, invasion and lung colonization in OSCC cells.

### MiR-99a does not influence cell morphology and subtly affect the expression of epithelial-mensenchymal transition (EMT)-related proteins and metalloproteinases

**Figure 3:**
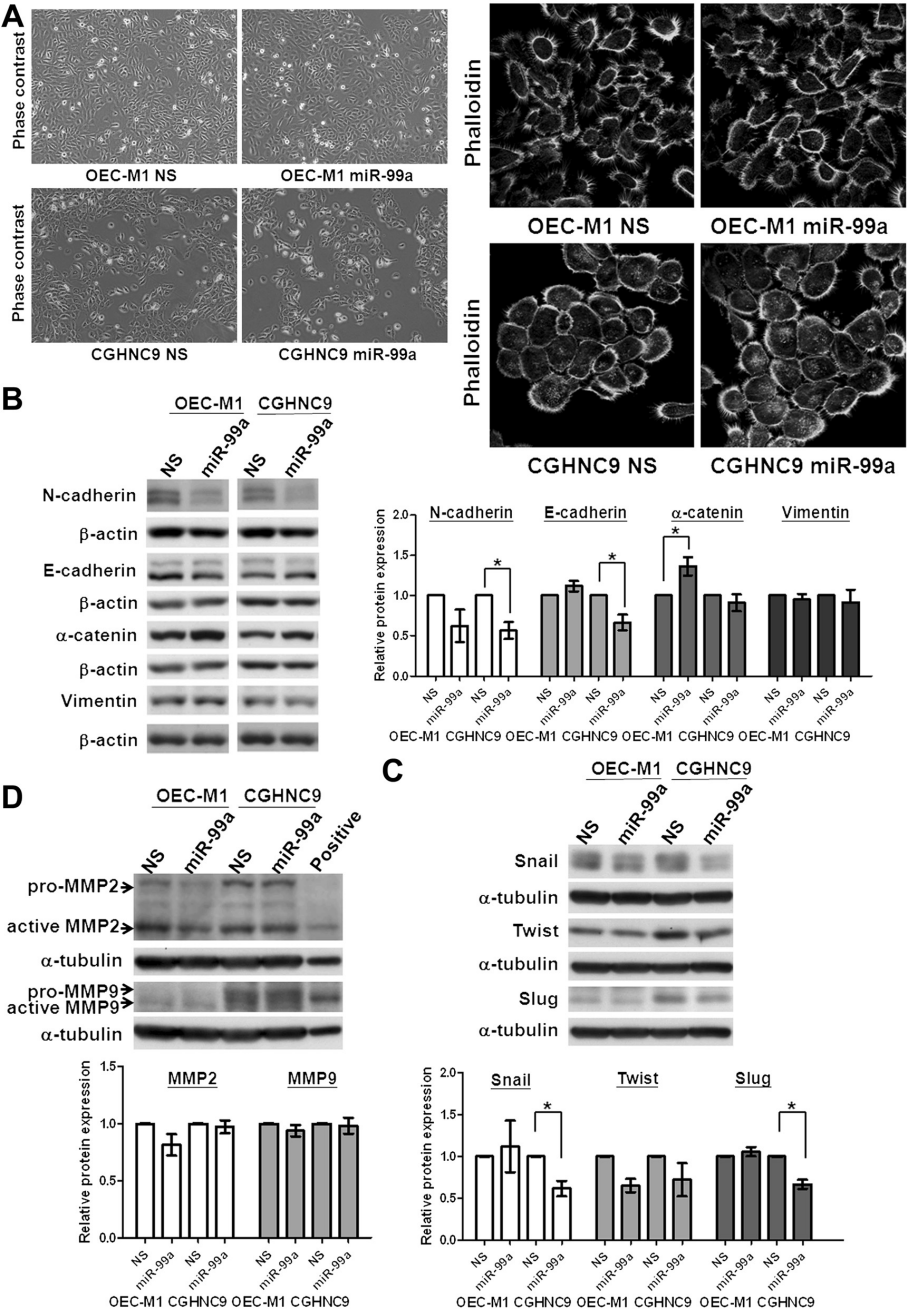
**Overexpression of miR-99a did not influence cell morphology and subtly affected the expression of EMT-related proteins and metalloproteinases. (A)** Ectopic miR-99a expression had no obvious effects on cell morphology when OEC-M1 and CGHNC9 cells with ectopic miR-99a expression (OEC-M1 miR-99a and CGHNC9 miR-99a) compared with their corresponding controls with lentiviral non-silencing microRNA (OEC-M1 NS and CGHNC9 NS) using phase contrast microscopy with 100× magnification (left panel). Cytoskeleton F-actin proteins were stained with Alex Fluro 488 phalloidin and viewed under fluorescence microscope with 630× magnification (right panel, shown in grey mode). **(B)** Immunoblot analysis of epithelial (E-cadherin and α-catenin) and mesenchymal (N-cadherin and vimentin) proteins in OEC-M1 NS, OEC-M1 miR-99a, CGHNC9 NS, and CGHNC9 miR-99a cells. **(C)** Immunoblot analysis of EMT-related transcription factors, including Snail, Slug, and Twist proteins in OEC-M1 NS, OEC-M1 miR-99a, CGHNC9 NS and CGHNC9 miR-99a cells. **(D)** Immunoblot analysis of pro- and active forms of metalloproteinase 2 (MMP2) and metalloproteinase 9 (MMP9) in OEC-M1 NS, OEC-M1 miR-99a, CGHNC9 NS, and CGHNC9 miR-99a cells. The protein levels were normalized against an internal control β-actin or α-tubulin. Ratios were determined by dividing the normalized protein levels in miR-99a expressing cells with that in control cells. The means of ratio in the graphs were measured by averaging the ratios from independent blots. Bar, SE; *p < 0.05.

Tumor cell migration is often associated with reorganization of the actin cytoskeleton and the occurrence of an EMT. We thus evaluated the effects of miR-99a on cell morphology and the EMT process. Analysis of cell morphology using light microscopy and fluorescent confocal microscope by phalloidin staining and immunoflurescence with anti-α-tubulin, E-cadherin and focal adhesion kinase (FAK) did not reveal any gross differences between miR-99a expressing cells and vector controls (Figure 3A and Additional file 2: Figure S1). Furthermore, results from immunoblot analysis showed that ectopic miR-99a expression slightly increased the expression of α-catenin, an epithelial protein in OEC-M1 cells, and reduced the expression of N-cadherin, a mesenchymal marker in CGHNC9 cells (Figure 3B). Ectopic miR-99a expression partially reduced the levels of EMT-related transcription factors, such as Snail, and Slug in CGHNC9 cells (Figure 3C). The expression of metalloproteinase 2 (MMP2) and 9 (MMP9) did not show a markedly reduction in cells with ectopic miR-99a expression (Figure 3D). Ectopic miR-99a expression, therefore, did not markedly influence the expression of the EMT-related proteins and transcription factors as well as metalloproteinases.

### MiR-99a negatively regulates IGF1R protein levels and reduces IGF1R signaling

**Figure 4:**
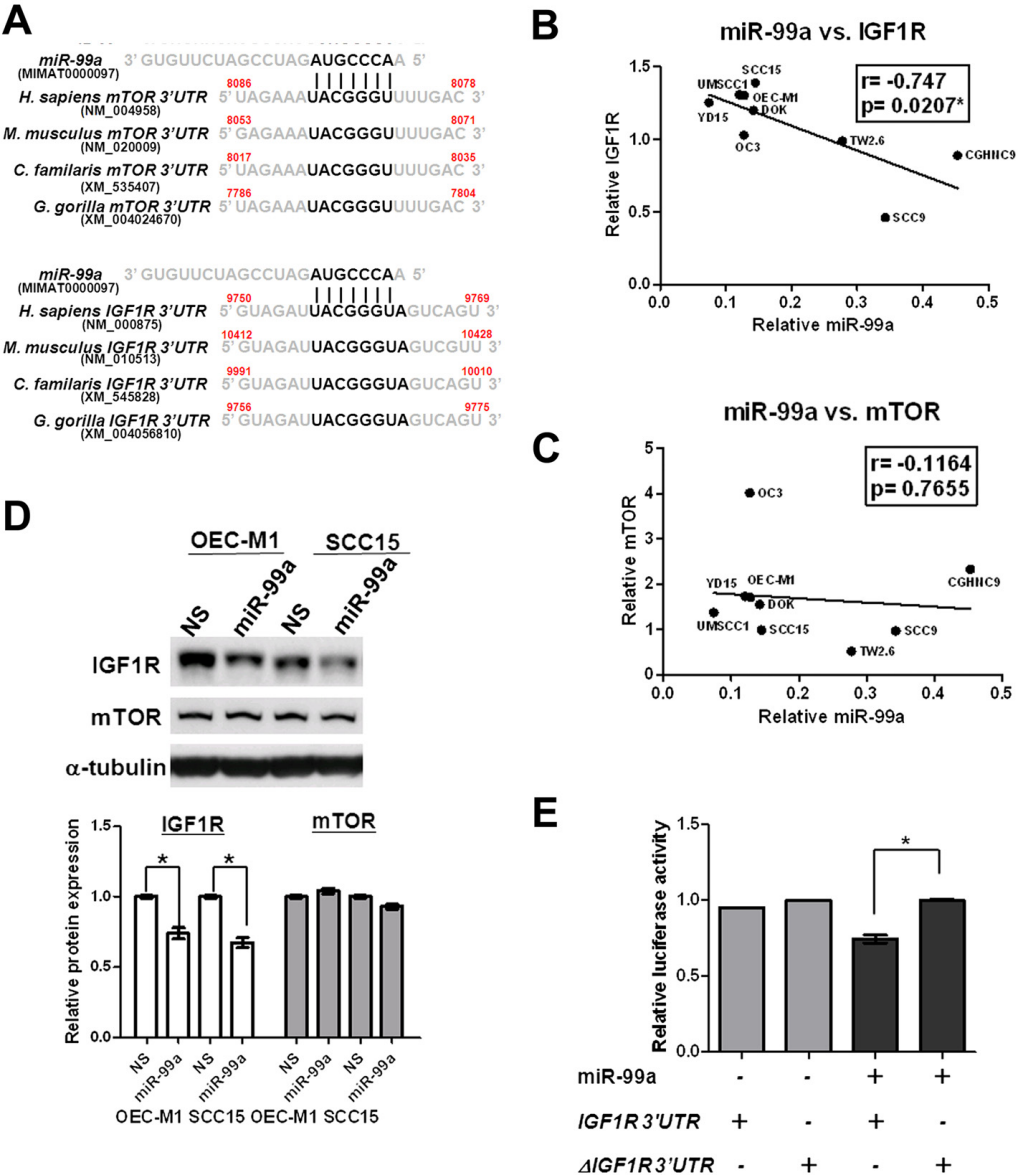
**MiR-99a targeted IGF1R in OSCC cells. (A)** Comparison of nucleotides in the miR-99a seed sequence and the targets, mTOR (upper panel) and IGF1R (lower panel), within the 3′UTR among species. **(B)** Significant negative correlation between the expression of miR-99a and IGF1R protein in OSCC cell lines (r = −0.747, p = 0.0207) by Pearson correlation. Average amounts of IGF1R protein were determined in two independent blots. *p < 0.05. **(C)** No significant correlation between expression of miR-99a and mTOR protein in OSCC cell lines by Pearson correlation (r = −0.1164, p = 0.7655). Average amounts of mTOR proteins were determined in two independent blots. **(D)** Immunoblot analysis of IGF1R and mTOR expression in OEC-M1 and SCC15 cells with ectopic miR-99a expression (OEC-M1 miR-99a and SCC15 miR-99a) or non-silencing microRNA expressing controls (OEC-M1 NS and SCC15 NS). The protein levels were normalized against an internal control α-tubulin. Ratios were determined by dividing the normalized protein levels in miR-99a expressing cells with that in non-silencing microRNA expressing controls. The means in the lower graph were measured by averaging the ratios from independent blots. Bar, SE; *p < 0.05. **(E)** Ectopic miR-99a expression suppressed the expression of the Renilla luciferase reporter gene containing the wild type miR-99a binding sequence in IGF1R 3′UTR (IGF1R 3′UTR) in OEC-M1 miR-99a cells. The luciferase activity of the construct with the deleted miR-99a binding site for IGF1R 3′UTR (ΔIGF1R 3′UTR) was assigned as 1. Bar, SE; *p < 0.05.

We further investigated if the specific binding of miR-99a caused the miR-99a-mediated reductions in expression of EMT-related protein, transcription and metalloproteinase by evaluating the correlation between miR-99a expression and the candidate gene expression using miRWalk for prediction (Additional file 1: Table S2) [26]. Results excluded this possibility and indicated that the components of the IGF1R signaling pathway, including mTOR and IGF1R, could be the potential targets of miR-99a (Additional file 1: Table S2). When analyzing the human sequences for interspecies homology, we observed that the miR-99a target sequences of IGF1R or mTOR are highly conserved among species (Figure 4A). Using gene expression analysis (GSE37991) of Taiwan OSCC tissues, we detected a >2-fold increase in IGF1R mRNA in 22/40 OSCC tissue samples in comparison with their corresponding adjacent nontumorous tissues (Additional file 3: Figure S2A). By immunoblot assay, we demonstrated up-regulated IGF1R expression in most of OSCC cells in comparison with HOK cells (Additional file 3: Figure S2B). The data indicated that IGF1R might play an important role in oral tumorigenesis. We then used Pearson correlation to determine whether IGF1R and mTOR are the possible targets of miR-99a, comparing miR-99a expression with the levels of IGF1R and mTOR protein in OSCC cells. We observed a significant negative correlation between miR-99a expression and levels of IGF1R protein (r = −0.747, p = 0.0207) (Figure 4B) but no such correlation between miR-99a expression and levels of mTOR protein in OSCC cells (r = −0.1164, p = 0.7655) (Figure 4C). This suggested the inverse correlation between the expression of miR-99a and that of IGF1R protein. To verify the targets of miR-99a, we investigated the mRNA and protein levels of IGF1R and mTOR in miR-99a expressing OSCC cells (Additional file 4: Figure S3 and Figure 4D). Results from Western blot analysis further showed that ectopic miR-99a expression decreased IGF1R expression, but not mTOR expression in OSCC cells (Figure 4D). To determine whether IGF1R mRNA is the specific binding target of miR-99a, we used the clones of the human IGF1R 3′UTR fragment containing the wild type or mutant miR-99a binding sequence in the region downstream of the Renilla luciferase reporter gene. In OEC-M1 cells with ectopic miR-99a expression, the luciferase activity of cells expressing wild type IGF1R 3′UTR was reduced 0.75 ± 0.03-fold in comparison with cells expressing the mutant seed region of miR-99a in the IGF1R 3′UTR (Figure 4E). However, in OEC-M1 control cells with low miR-99a expression, the luciferase activity of cells expressing wild type or mutant IGF1R 3′UTR remained unchanged. These data indicated that miR-99a negatively regulates IGF1R expression by directly targeting the 3′UTR of IGF1R mRNA and reducing IGF1R protein levels. To determine whether ectopic IGF1R expression was sufficient to rescue the miR-99a-mediated inhibition of migration and invasion, IGF1R was transiently expressed in miR-99a expressing OEC-M1 cells and confirmed by immunoblot assay (Additional file 5: Figure S4A). We observed that the migration and invasion activities were rescued by ectopic IGF1R expression in miR-99a expressing OEC-M1 cells, indicating that IGF1R was involved in miR-99a-mediated reduction of migration and invasion (Additional file 5: Figure S4B).

### The IGF1R signaling pathway negatively regulates miR-99a expression

**Figure 5:**
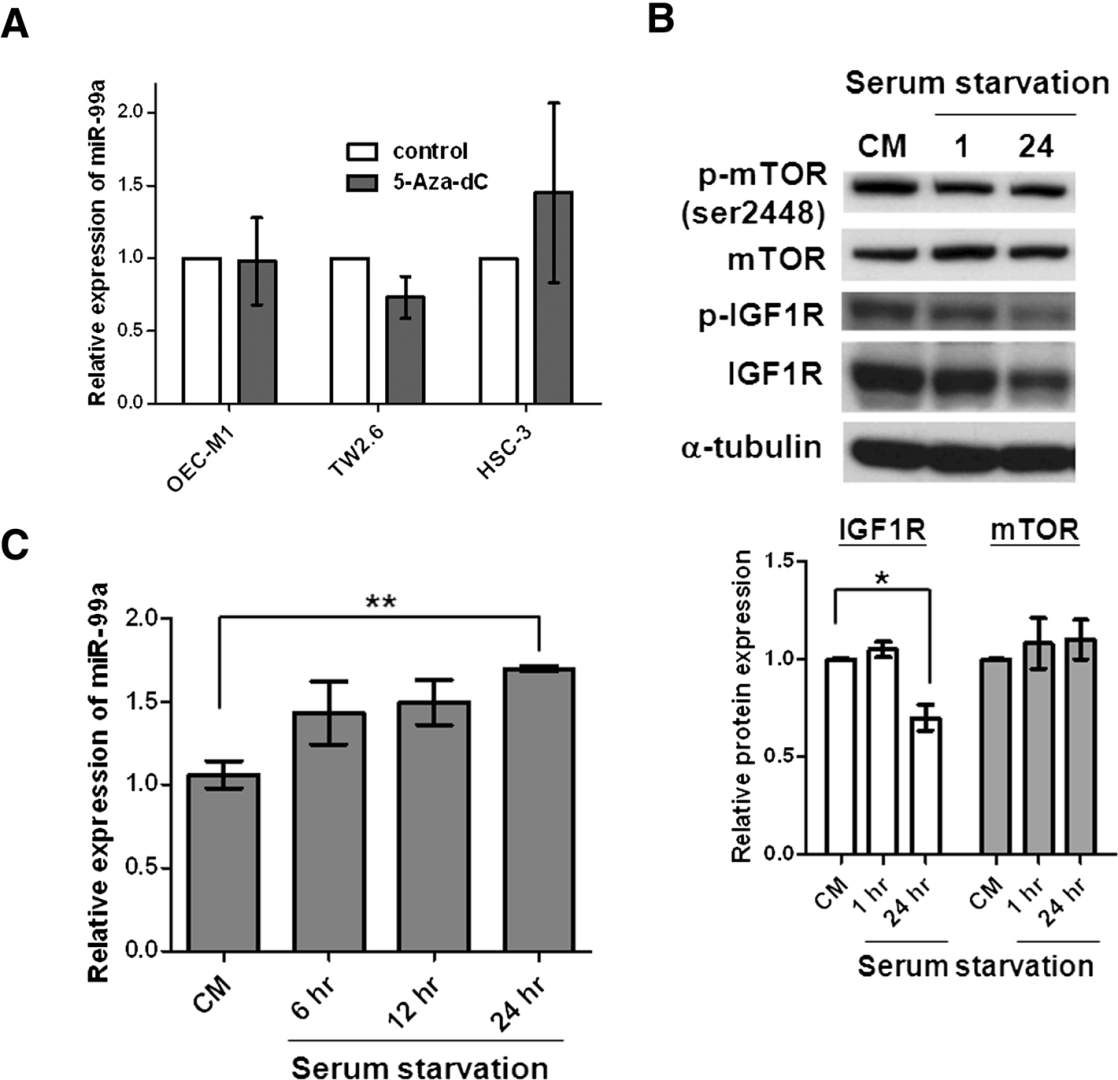
**Regulation of miR-99a expression in different culture conditions. (A)** The levels of miR-99a expression in OEC-M1, TW2.6, and HSC3 oral cancer cells treated with 5 μM 5-Aza-dC for 96 hours were determined using qRT-PCR analysis. Expression of miR-99a was normalized against an endogenous control U6. Data from 5-Aza-dC-treated OSCC cell lines were normalized to those from untreated controls. **(B)** Immunoblot analysis revealed reduced levels of total and phosphorylated IGF1R protein in non-silencing microRNA expressing controls (OEC-M1 NS) with serum starvation. Ratios were determined by dividing the protein levels in serum-starved cells with that in complete medium (CM). The means in the lower panel were measured by averaging the ratios from independent blots. Bar, SE; *p < 0.05. **(C)** MiR-99a expression increased in serum starvation in a time-dependent manner. Expression of miR-99a was normalized against an endogenous control U6. The relative expression of miR-99a was determined by normalizing the expression of miR-99a in OEC-M1 NS cells in serum starvation to that in complete medium (CM). Bar, SE; **p < 0.01.

To elucidate the mechanisms underlying the downregulation of miR-99a, we first investigated the possibility of genetic methylation, which typically occurs at CpG sites and inactivates gene transcription. We computationally mapped the CpG islands upstream of the miR-99a gene but detected no CpG-enriched region (data not shown). We then treated 3 OSCC cell lines that express low levels of miR-99a with 5-aza-deoxygcitidine (5-Aza-dC), a methylation inhibitor. However, this treatment did not increase miR-99a expression (Figure 5A). These data suggested that promoter methylation did not cause the downregulation of miR-99a.

The levels of total and phosphorylated IGF1R protein reduced in serum starvation, suggesting the possibility of upregulation of miR-99a in serum-free conditions (Figure 5B). To verify this, we analyzed the levels of miR-99a in cells cultured in serum-free medium for different duration, observing that a 1.7-fold increase of miR-99a expression was detected in cells subjected to serum starvation for 24 hours and the expression of miR-99a increased significantly in serum starvation in a time-dependent manner (Figure 5C). Collectively, these data suggested the upregulation of miR-99a expression following the removal of supplied growth factors.

**Figure 6:**
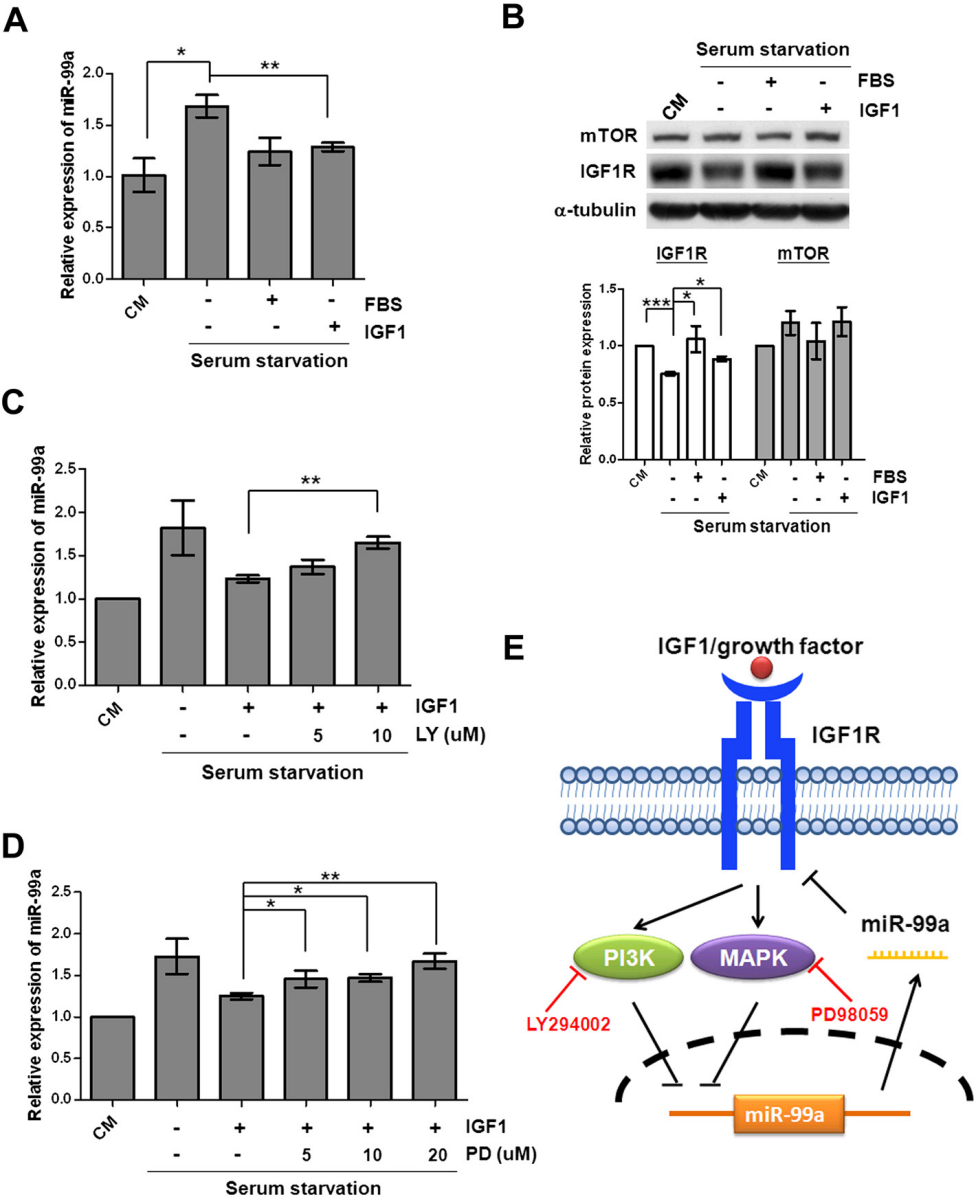
**Negative regulation of miR-99a expression by the IGF1R signaling pathway. (A)** Downregulation of miR-99a in response to IGF1 treatment. After serum starvation, cells were treated with vehicle, 10% FBS or 10 nM IGF1. **(B)** Immunoblot analysis revealed increased levels of IGF1R protein following IGF1-induced downregulation of miR-99a. After serum starvation, cells were treated with vehicle, 10% FBS or 10 nM IGF1. The protein levels were normalized against an internal control α-tubulin. Ratios were determined by dividing the normalized protein levels in OEC-M1 cells with different conditions to that in complete medium (CM). The means in the lower panel were measured by averaging the ratios from independent blots. **(C)** The PI3K inhibitor LY294002 and **(D)** MAPK kinase inhibitor PD98059 suppressed the IGF1-induced downregulation of miR-99a. After serum starvation, cells were treated with vehicle, 10 nM IGF1, or combination of LY294002/PD98059 and IGF1. Expression of miR-99a was normalized against an endogenous control U6. The relative expression of miR-99a was determined by normalizing the expression of miR-99a in OEC-M1 cells in different conditions to that in cells in complete medium (CM). Bar, SE; *p < 0.05; **p < 0.01; ***p < 0.001. **(E)** Proposed model of the relationship between the activation of the IGF1R signaling pathway and miR-99a expression. The activation of the IGF1R signaling pathway initiated the inhibitory signal leading to decreased miR-99a expression. Following the suppression of the IGF1R signaling pathway by inhibitors, or its silencing by the removal of stimulation, we observed increased miR-99a expression because of the removal of the inhibitory signal. This reciprocal regulation of miR-99a and IGF1R signaling augmented the activation of the IGF1R signaling pathway in response to IGF1 stimulation and accelerated its inactivation following the removal of stimulation.

Following the retreatment of cells with serum or IGF1 after serum starvation, we observed reduced expression of miR-99a compared with cells in serum-free conditions (Figure 6A). This suggested that serum or IGF1 regulates the expression of miR-99a. Results from immunoblot analysis indicated that IGF1R protein was reduced in serum starvation conditions, whereas IGF1R protein was significantly increased following serum or IGF1 treatment (Figure 6B). These data indicated the negative regulation of miR-99a expression by serum or IGF1 stimulation. To verify whether the regulation of miR-99a is mediated by activation of IGF1R signaling pathway, the data showed that cells displayed the decreased phosphorylation levels of AKT and MAPK upon treatment with the LY294002 and PD98059 inhibitors for downstream of IGF1R signaling pathways, PI3K and MAPK kinase, respectively (Additional file 6: Figure S5). We found that the inhibitors LY294002 and PD98059 eliminated IGF1-induced downregulation of miR-99a in a dose-dependent manner (Figures 6C and 6D). Additionally, levels of miR-99a were decreased in non-silencing miRNA expressing OEC-M1 cells with ectopic expression of IGF1R (Additional file 5: Figure S4C). Overall, these findings suggested the negative regulation of miR-99a expression by the IGF1R signaling pathway and the presence of a reciprocal regulation for the mutual regulation of miR-99a expression and IGF1R protein levels.

## Discussion

Previous studies used miRNA microarray and qRT-PCR analyses to characterize the miRNA expression profiles in oral cancer cell lines and clinical samples of different stages [27,28]. Their findings suggested the dysregulation of miRNAs in the initiation and progression of oral cancers. Similar to the results from a study by Wong et al. [14], our data showed that miR-99a is frequently downregulated in OSCC cell lines and tissues, especially in OSCC patients with lymphovascular invasion (Figure 1 and Additional file 1: Table S1). This suggested that the downregulation of miR-99a might play a critical role in invasion and metastasis of OSCC and supported evidence that ectopic miR-99a expression inhibits cell migration, invasion and lung colonization in OSCC cells (Figures 2D-G). Li et al. reported that low miR-99a expression in hepatocellular carcinoma (HCC) tissues was associated with worse prognosis in HCC patients [10]. However, in this study, we observed insignificant correlation between miR-99a expression and most of clinicopathological parameters (Additional file 1: Table S1).

The biological roles of miR-99a in oral tumorigenesis remain unclear. Previous studies described that ectopic miR-99a expression slowed keratinocyte growth and liver cancer cell growth by blocking the cell cycle at the G1/S transition [10,29]. In OEC-M1 and CGHNC9 cells, ectopic miR-99a expression had no significant effects on cell growth (Figures 2B and C), cell cycle (Additional file 7: Figure S6A) and cell morphology (Figure 3A and Additional file 2: Figure S1) even though ectopic expression of miR-99a did subtly affect the expression of cell cycle-related molecules (Additional file 7: Figure S6B) and EMT-related proteins (Figure 3). However, ectopic miR-99a expression decreases cell migration, invasion and lung colonization (Figures 2D-G). These observations suggested that the diverse biological functions of miR-99a may be cell type- and context- dependent. Because of low miR-99a expression in all tested OSCC cells, we were unable to observe any significant knockdown of miR-99a in OSCC cells in response to anti-miR-99a expression (data not shown).

With bioinformatic prediction and experimental validation using microarray and polyribosomal loading analysis, Sun et al. identified that IGF1R and mTOR are the direct targets of miR-99a [9]. In our study, the expression of miR-99a correlated inversely with IGF1R, but not mTOR protein expression (Figures 4B and 4C). Ectopic expression of miR-99a decreased the levels of IGF1R protein (Figure 4D and Additional file 4: Figure S3), suggesting that IGF1R, but not mTOR, is a specific target of miR-99a in OSCC cells. However, it remains possible that miR-99a might functionally target mTOR in other cell types or in different biological systems [12]. It is, therefore, possible that other molecules or signaling pathways influenced by miR-99a could be involved in OSCC pathogenesis, and that some of them have yet to be identified.

Insulin-like growth factor 1 receptor is overexpressed in several malignancies and plays a crucial role in promoting cell proliferation and metastasis [30]. Our results demonstrated that miR-99a expression decreases IGF1R protein levels by directly targeting the 3′UTR of IGF1R mRNA (Figure 4E). Previous studies have identified that IGF1R is also a specific target of miR-145 in colon cancer cells [31], miR-122 in liver cancers [32], miR-7 in tongue squamous cell carcinoma cells [28], miR-375 in esophageal squamous cell carcinoma [33], and let-7c in regulation of glucose metabolism [34]. A recent study showed that the administration of tumor-suppressive miRNA to mice could effectively suppress tumorigenesis in liver cancer without causing toxicity [35]. This indicated the possibility of using miRNA to target IGF1R in cancer therapy.

Although previous studies identified reduced miR-99a expression in several types of tumors [8,9,11,12,14,36-38], the detailed mechanisms underlying its repression remain unclear. As previously reported, epigenetic mechanisms, including DNA methylation and histone modification [39], chromosome deficiency [40], and transcriptional regulation [41] can influence the downregulation of miRNA. Yamada et al. showed that the gene encoding miR-99a, located in chromosome 21q21, is frequently deleted in lung cancers [36]. In this study, we examined 40 paired tissue samples from OSCC patients for chromosome deletions using array comparative genomic hybridization and failed to identify any obvious deletion in chromosome 21q21 (data not shown). We then considered the hypermethylation of the CpG islands surrounding miRNA genes as markers for the investigation of epigenetically silenced miRNAs. However, reactivated expression of miR-99a was undetectable in oral cancer cells after treatment with 5-Aza-dC (Figure 5A). Similarly, Li et al. suggested that there was little evidence of the contribution of methylation to downregulation of miR-99a [10]. These findings indicated that promoter methylation did not cause the downregulation of miR-99a.

Reduced endogenous miR-99a in OSCC cells retreated with IGF1 after serum starvation (Figure 6A), increased miR-99a expression in cells treated with PI3K or MAPK kinase inhibitors (Figures 6C and 6D), and a reduced level of miR-99a in OSCC cells provided further evidence of the regulation of levels of miR-99a by IGF1R signaling. We proposed a model to describe the combining of the functions of miR-99a and IGF1R to achieve a maximal effect in OSCC cells in response to IGF1R activation (Figure 6E). Activation of the IGF1R signaling pathway initiated the inhibitory signal for miR-99a expression. Decreased miR-99a expression then increased the levels of IGF1R protein. Inhibition of the IGF1R signaling pathway by depletion of growth factors or specific chemical inhibitors increased miR-99a expression because of the loss of the inhibitory signal. This then reduced the levels of IGF1R protein. This reciprocal regulation, therefore, maximizes the activation of the IGF1R signaling pathway in OSCC cells in response to IGF1 stimulation. Due to tumor heterogeneity, however, our results indicated a reciprocal regulation that mutually regulates the levels of miR-99a and IGF1R in a part of OSCC cells. Different from our hypothesis, Lerman et al. described that the activation of IGF1 signaling increased the expression of miR-99a, which then repressed the expression of IGF1R in psoriatic skin [29]. Huang et al. showed that IGF1 significantly accelerated the upregulation of miR-133, which targeted IGF1R during skeletal myogenesis [42]. Both studies described the reciprocal regulation of miRNA and IGF1R during proliferation and differentiation in normal tissues. Collectively, this reciprocal regulation is potentially involved in a number of genetic pathways involving miRNA, and might enhance the functionality and robustness of gene networks [43]. Other previous studies described similar findings involved in the regulation of let-7 g and lectin-like oxidized LDL receptor-1 (LOX-1) [44]; let-7 and Fas [45]; ZEB1-SIP and the miR-200 family [46].

The mechanisms for the negative regulation of miR-99a by the IGF1R signaling pathway remain unclear. Several studies described the positive correlation between the expression of mature miR-99a and primary miR-99a and that of their host gene C21orf34/LINC00478 in liver and prostate cancer tissues, suggesting the possible cotranscription of miR-99a with C21orf34 [10,47]. Willimott et al. reported that stromal cell contact induced the expression of the miRNA cluster miR-99a/let-7c/miR-125b, indicating the involvement of transcriptional regulation in the induction of this miRNA cluster [48]. Future investigation to evaluate the possible promoter regions and to identify the transcription factors binding to the region upstream of the miR-99a gene is, therefore, warranted.

Collectively, we demonstrated that miR-99a is frequently down-regulated and functions as a tumor metastasis suppressor in OSCC cells. Also, miR-99a mutually regulates its own target, IGF1R expression within a reciprocal regulation, suggesting that the possibility of miR-99a for targeting IGF1R in cancer therapy.

## Materials and methods

### Clinical samples and patient characteristics

Paired tumor specimens and their adjacent nontumorous epithelia were derived from 40 primary OSCC patients who received curative surgery from 2002 to 2009 at National Cheng Kung University Hospital. Fresh frozen tissues were preserved in liquid nitrogen until use. Clinical parameters, including age, sex, social history, pathological features, and TMN stage, were retrospectively collected by reviewing patients’ charts. The study protocol was approved by the Institutional Human Experiment and Ethics Committee. Informed consent was obtained from each patient.

### Oral squamous cell carcinoma cell lines

Human oral keratinocytes (HOK) were purchased from ScienCell Research Laboratories and cultured in an oral keratinocyte medium (OKM; ScienCell Research Laboratories, Carlsbad, CA, USA) according to the manufacturer’s instructions. OSCC cell lines, including CGHNC9 [28], OC3 [49], OEC-M1 [50], TW2.6 [51], FaDu [52], KB [53], SCC-4, SCC15 [54], SCC9, SCC25 [55], UT-MUC-1 [56], YD-15 [57], DOK [58], Tu183 [59], UMSCC1 [60] and HSC3 [61], were cultured at 37°C in a 5% CO_2_ atmosphere within 3 months of resuscitation from the frozen aliquots, with lower than 20 passages in each experiment.

### Quantification of miRNA

Total RNA molecules were polyadenylated and reverse transcribed using poly (A) polymerase and MMLV reverse transcriptase in a Mir-X™ miRNA First-Strand Synthesis kit (Clontech, Madison, WI, USA) according to the manufacturer’s manual. Real-time quantitative polymerase chain reactions were performed using a Fast SYBR® Green Master Mix (Applied Biosystems, Foster City, CA, USA) for amplification. Specific miRNA sequences in the cDNA were quantified using miRNA-specific sequences as 5′ primers. The forward primer used for miR-99a was 5′ AACCCGTAGATCCGATCTTGTG. The U6 was used as a reference for miRNA quantification and was supplied in the kit. All amplifications were performed in triplicate and values were normalized to an endogenous control U6. The relative expression of miRNA was normalized to that of the control in each experiment.

### Immunoblot analysis

Immunoblot assays were performed as described previously [62]. Primary antibodies were used as followed: anti-E-cadherin (610182, BD, San Jose, CA, USA), anti-α-catenin (610193, BD), anti-N-cadherin (610920, BD), anti-Vimentin (MS-129-P0, Thermo Scientific, Cheshire, UK), anti-Twist (sc-15393, Santa Cruz, Santa Cruz, CA, USA), anti-Snail (3895, Cell Signaling, Danvers, MA, USA), anti-Slug (AP2053a, Abgent, San Diego, CA, USA), anti-MMP2 (#4022, Cell Signaling), anti-MMP9 (#2551-1, Epitomics, Burlingame, CA, USA), anti-phospho-mTOR (Ser2448) (#5536, Cell Signaling), anti-mTOR (#2972, Cell Signaling), anti-phospho-IGF1 Receptor β (Tyr980) (#4568, Cell Signaling), anti-IGF1 Receptor β (#3018, Cell Signaling), anti-p21 (#2946, Cell Signaling), anti-p27 (#2552, Cell Signaling), anti-cyclin D1 (#2926, Cell Signaling), anti-cyclin E (sc-247, Santa Cruz), anti-β-actin (sc-1615, Santa Cruz) and anti-α-tubulin (MS-581-P0, Thermo Scientific). Protein levels were determined by measuring the intensity of bands on the blots using Image J (National Institutes of Health, Bethesda, Maryland, USA). Protein levels were normalized against an internal control β-actin or α-tubulin. The ratio was determined by dividing the normalized protein levels in expressing cells with that in control cells. The mean of ratio was obtained by averaging the ratios from several independent blots.

### Cell proliferation

Cell proliferation was measured using 3-[4,5-dimethylthiazol-2-yl]-2,5-diphenyl tetrazolium bromide (MTT) assay as described previously [63]. 10^3^ cells were seeded onto 96-well plate and measured their growth by CellTiter 96 Aqueous Non-Radioactive Cell Proliferation assay (Promega, Madison, WI, USA) for 4–5 days according to the manufacturer’s instructions. The proliferation curves were determined by calculating the mean value of absorbance measurement at 490 nm using a 96-well plate reader.

### Migration and invasion assay

Migration and invasion assays were performed using transwells as described previously [62]. Briefly, cells in 0.5 mL of serum-free medium were plated into the inserts (Matrigel-coated inserts for invasion assay). Inserts were placed in wells with 0.75 mL of complete medium containing 10% FBS as a chemoattractant. After culture for 20 to 24 hours at 37°C, cells were fixed with methanol for 8 min and stained with Giemsa regent (Sigma, St Louis, MO, USA). Cells on the upper sides of the inserts were removed with a cotton swab, and the insert membranes were cut and mounted on glass slides. The numbers of migrated or invaded cells on the membranes were determined by counting the cell numbers in the field at 100X magnification under light-field microscope. The relative migration/invasion activity was measured by normalizing the mean of total migrated/invaded cells per insert in overexpressing cells to that in the corresponding controls. The data shown represent the average of at least 3 repeated experiments.

### Lung colonization assay

Lung colonization assay was performed as described previously [63]. All animal studies were followed by the guidelines for the Care and Use of Laboratory Animals of National Health Research Institutes, Taiwan. The protocol was approved by the Institutional Animal care and Use Committee of National Health Research Institutes (Protocol No: NHRI-IACUC-100047-A). All efforts were made to minimize suffering. Briefly, 10^5^ cells suspended in phosphate buffered saline (PBS) were injected into nude mice (National Laboratory Animal Center, Taiwan) via tail vein injection. The whole lungs were harvested for paraffin embedding, sectioning, and histological examination after H&E staining (Pathology Core, National Health Research Institutes, Taiwan). 6 animals were included in each group. The number of tumor nodules in every lung section was counted under the light-field microscope. The colony number/per lung section was determined by averaging the numbers of tumor nodules from independent lung sections.

### Immunofluorescence

Cultured cells on slips were fixed and followed the protocols as previous described [62]. After incubation with Alexa Fluro 488 phalloidin (1:200, Molecular Probes, Ungene, OR, USA), anti-β-tubulin (MS-581-P0, Thermo Scientific), anti-E-cadherin (610182, BD) and anti-FAK (sc-557, Santa Cruz), the slips were mounted with antifade onto the slides and viewed under a fluorescence confocal microscope.

### Lentiviral infection of miR-99a

The expression construct of miR-99a and non-silencing microRNA were kindly donated by Dr Michael Hsiao, Academic Sinica, Taiwan and transfected into the packaging cell line 293FT, along with pMD.G and pCMVΔR8.91 plasmids, using the Polyjet transfection reagent (SignaGen Lab, Ijamesville, MA, USA). After 48-hour incubation, viral supernatants were transferred to target cells and infected cells were cultured in the presence of different concentrations of puromycin (Calbiochem, La Jolla, CA, USA) depending on the cell lines.

### Luciferase reporter assay

The IGF1R (nucleotides 9543 to 9833) 3′UTR sequence (IGF1R 3′UTR) and deletions in the 3′UTR-predicted miR-99a binding sites (ΔIGF1R 3′UTR) were cloned in psiCHECK-2 vector (Promega) and donated by Dr Enzo Lalli [8]. The OEC-M1 cells with ectopic miR-99a (OEC-M1 miR-99a) or non-silencing microRNA expression (OEC-M1 NS) were transfected in 6-well plates according to the manufacturer’s protocol. Rellina and firefly luciferase assays were performed using the Dual-Luciferase Reporter® Assay System (Promega), using a luminometer for measurement of luminescence. In each sample, the luciferase activity was determined by normalizing the Renilla luciferase activity to the firefly luciferase activity. The relative luciferase activity was determined by normalizing the activity in cells with wild type 3′UTR expression (IGF1R 3′UTR) to that in cells with the mutant 3′UTR (ΔIGF1R 3′UTR). Experiments for each construct were repeated 2 to 4 times.

### Transient expression of Insulin-like growth factor I receptor

Expression construct of IGF1R was kindly obtained from Dr. Lu-Hai Wang, National Health Research Institutes, Taiwan. The plasmids were transiently transfected into cells using the Polyjet transfection reagent (SignaGen Lab). After 48-hour incubation, cells were collected to perform the subsequent experiments.

### Prediction of miR-99a targets

Using miRWalk http://(http://www.umm.uni-heidelberg.de/apps/zmf/mirwalk/), a comprehensive database with eight established programs, including DIANA-microT (version 3.0), miRanda (August 2010), miRDB (April 2009), PicTar (March 2007), PITA (August 2008), RNA22 (May 2008), RNAhybrid (version 2.1) and Targetscan (version 5.1), miR-99a targets were predicted in 10 datasets (DIANA-mT, miRanda, miRDB, miRwalk, RNAhybrid, PICTAR4, PICTAR5, PITA, RNA22, TargetScan) for binding sites on 3′UTR of mRNA. For correlations, the expression of miR-99a and interested mRNA was analyzed in 40 pairs of OSCC patients (GSE37991) using Pearson correlation.

### Drug treatment

Cells were seeded and treated with 5-Aza-dC (5 μM; Sigma). After incubating for 96 hours with a change of culture medium every 24 hours, cells were collected for further analysis.

Cells were seeded overnight and refreshed with serum-free medium, then subjected to serum starvation for 12 hours. After starvation, cells were treated with vehicle, 10% fetal bovine serum (FBS) or 10 nM IGF1 (Sigma) for different durations. For experiments involving inhibition of signaling pathways, cells were cultured in the presence of PD98059 (Sigma) or LY294002 (Sigma), specific inhibitors of MAPK kinase and PI3K, respectively, for 1 hour prior to IGF1 stimulation.

### Statistical analysis

Data are presented as the mean  ±  standard error of the mean (SE) from at least 2 independent experiments. For comparisons between 2 groups, the differences between the groups were analyzed using 2-tailed student’s *t* test. Linear regression, Pearson correlation, and Spearman correlation were used to evaluate the correlation between 2 variants. Analysis was performed using GraphPad Prism version 5.01 (GraphPad Software, La Jolla, CA, USA). For all comparisons, p < 0.05 was considered statistically significant.

## Abbreviations

OSCC: Oral squamous cell carcinoma; miRNA: microRNA; miR-99a: microRNA-99a; IGF1R: Insulin-like growth factor 1 receptor; mTOR: mammalian target of rapamycin; IGF1: Insulin–like growth factor 1; MAPK: Mitogen-activated protein kinase; PI3K: Phosphatidylinositol 3-kinase; AKT/PKB: Protein kinase B; 5-Aza-dC: 5-aza-2′-deoxycytidine; qRT-PCR: quantitative reverse transcription-polymerase chain reaction; 3′UTR: 3′ untranslated region.

## Competing interests

The authors declare that they have no conflict of interest.

## Authors’ contributions

YCY carried out the molecular and cellular biology studies and helped to draft the manuscript. SGS performed the bioinformatic analysis and qRT-PCR. HCC performed Western blots. YMH performed the immunofluorescence. JRH collected the information of OSCC patients and helped to draft the manuscript. JYC participated in design of the study and collection of OSCC patients. WCH helped to draft the manuscript. CTL participated in establishment of cell lines and helped to draft the manuscript. AJC participated in establishment of cell lines. YCL participated in establishment of cell lines. YWC participated in the design of the study and coordination and drafted the manuscript. All authors read and approved the final manuscript.

## Supplementary Material

Additional file 1: Table S1 Correlation between clinical parameters and relative expression of miR-99a in 40 oral squamous cell carcinoma (OSCC) patients^#^. **Table S2.** Prediction of candidate targets of miR-99a by miRWalk and correlations between miR-99a expression and candidate gene expression in 40 pairs of oral squamous cell carcinoma (OSCC) (GSE37991) by Pearson correlation. Click here for file

Additional file 2: Figure S1 Over-expression of miR-99a did not change cell morphology. (A) Ectopic miR-99a did not change cell morphology in miR-99a expressing OEC-M1 (OEC-M1 miR-99a) and CGHNC9 (CGHNC9 miR-99a) cells when compared with their non-silencing microRNA expressing controls, OEC-M1 NS and CGHNC9 NS under phase contrast microscopy with 400X magnification, respectively. (B) Immunofluorescence using anti-α-tubulin, (C) anti-E-cadherin and (D) anti-focal adherin kinase (FAK) showed similar patterns in OEC-M1 (OEC-M1 NS and OEC-M1 miR-99a) and CGHNC9 (CGHNC9 NS and CGHNC9 miR-99a) cells under fluorescent confocal microscope with 630X magnification (shown in grey mode). Click here for file

Additional file 3: Figure S2 Expression of IGF1/IGFR1 in OSCC tissues and cells. (A) The level of IGF1R mRNA was up-regulated in 22/40 (55%) of OSCC tissues with >2-fold increase by microarray analysis when compared with their corresponding nontumorous parts. Up-regulated IGF1 mRNA was not detectable in 40 pairs of OSCC tissues. (B) Immunoblot assay for detection of IGF1R protein in two independent batches of HOK and OSCC cells (upper panel). The protein levels were normalized against an internal control β-actin. Ratios were determined by dividing the normalized protein levels in OSCC cells with that in HOK cells. The mean of ratio in the graphs was measured by averaging the ratios from two independent blots (lower panel). Bar, SE. Click here for file

Additional file 4: Figure S3 Qunatification of IGF1R and mTOR mRNA in miR-99a expressing OSCC cells. Quantitative RT-PCR demonstrated the relative mRNA levels for IGF1R and mTOR in OEC-M1 and SCC15 cells with ectopic miR-99a expression (OEC-M1 miR-99a and SCC15 miR-99a) or non-silencing microRNA expressing controls (OEC-M1 NS and SCC15 NS). All amplifications were normalized to an endogenous β-actin control. The relative expression of mRNA in miR-99a expressing cells was normalized to that in non-silencing microRNA expressing controls. Bar, SE; ***, p < 0.001. Click here for file

Additional file 5: Figure S4 Figure S4 IGF1R rescued the inhibition of migration and invasion in miR-99a expressing OEC-M1 cells. (A) Protein levels of IGF1R expression were determined by Western blot in miR-99a expressing OEC-M1 (OEC-M1 miR-99a) cells and non-silencing microRNA expressing controls (OEC-M1 NS) with ectopic IGF1R expression. α-tubulin served as a loading control. (B) Representative data showed the relative migration/invasion activity of OEC-M1 NS and OEC-M1 miR-99a cells expressing IGF1R (OEC-M1 NS/IGF1R and OEC-M1 miR-99a/IGF1R) and their vector controls (OEC-M1 NS/VC and OEC-M1 miR-99a/VC). The relative migration/invasion activity was defined by normalizing the mean of migrated or invaded cells/per field in cells expressing IGF1R to that in OEC-M1 NS/VC. Bar, SE; *p < 0.1; ***p < 0.001. (C) Levels of miR-99a were determined by qRT-PCR in OEC-M1 NS cells with ectopic IGF1R expression. MiR-99a expression was normalized against an endogenous control U6. The relative expression of miR-99a was presented by normalizing miR-99a expression in OEC-M1 NS cells with ectopic IGF1R expression (OEC-M1 NS/IGF1R) to that in the controls (OEC-M1 NS/VC). Bar, SE; *** p < 0.001. Click here for file

Additional file 6: Figure S5 Activation of AKT and MAPK by IGF1 stimulation was inhibited upon treatment with the PI3K inhibitor LY294002 and MAPK kinase inhibitor PD98059, respectively. After serum starvation, cells were treated with vehicle, 10 nM IGF1, or combination of LY294002/PD98059 and IGF1. Immunoblot assay showed that levels of phosphorylated AKT and MAPK in IGF1-stimulated OEC-M1 cells were inhibited upon treatment with LY294002 and PD98059, respectively. Click here for file

Additional file 7: Figure S6 Ectopic miR-99a expression did not change cell cycle but subtly affected the expression of cell cycle-related proteins. (A) Ectopic miR-99a expression did not change the cell cycle in OEC-M1 and CGHNC9 cells using propidium iodide staining. (B) Immunoblot analysis of cell cycle-related molecules, including cyclin D, cyclin E, p21 and p27 in OEC-M1 and CGHNC9 cells with ectopic miR-99a expression (OEC-M1 miR-99a and CGHNC9 miR-99a) or non-silencing microRNA expressing controls (OEC-M1 NS and CGHNC9 NS). α-tubulin served as an internal control. Click here for file
