# Supplementary material for: Reciprocal regulation of MicroRNA-99a and insulin-like growth factor I receptor signaling in oral squamous cell carcinoma cells
Source: Mol Cancer. 2014 Jan 10;13:6. doi: 10.1186/1476-4598-13-6 (PMC3895693; doi:10.1186/1476-4598-13-6)
Supplement: Additional file 1: Table S1 — Correlation between clinical parameters and relative expression of miR-99a in 40 oral squamous cell carcinoma (OSCC) patients#. Table S2. Prediction of candidate targets of miR-99a by miRWalk and correlations between miR-99a expression and candidate gene expression in 40 pairs of oral squamous cell carcinoma (OSCC) (GSE37991) by Pearson correlation. [file 1476-4598-13-6-S1.doc]

**Additional file 1**

**Reciprocal Regulation of MicroRNA-99a and Insulin-like Growth Factor I Receptor Signaling in Oral Squamous Cell Carcinoma Cells**

Yi-Chen Yen, Shine-Gwo Shiah, Hsiao-Chien Chu, Yuan-Ming Hsu, Jenn-Ren Hsiao, Jang-Yang Chang, Wen-Chun Hung, Chun-Ta Liao, Ann-Joy Cheng, Ya-Ching Lu and Ya-Wen Chen

**Materials and methods**

**Quantitative reverse transcription-polymerse chain reaction (qRT-PCR)**

Total RNA was isolated from cell lines by Trizol reagents (Invitrogen) according to the manufacturer’s protocols. Two micrograms of RNA was reverse-transcribed into complementary DNA (cDNA) using Superscript first-strand cDNA synthesis system (Invitrogen) according to the manufacturer’s manual. The cDNA were amplified in a real-time PCR system (Applied Biosystems) using SYBR Green master PCR mix (Applied Biosystems). The primer sequences were used as follows: IGF1R forward primer: 5’-CTTGTACATTCGCACCAATGCT; IGF1R reverse primer: 5’ -CGATTAACTGAGAAGAGGAGTTCGA; mTOR forward primer: 5’-GGCTGATGGACACAAATACCAA; mTOR reverse primer: 5’ -TGGTCCCCGTTTTCTTATGG; β-actin forward primer: 5`-TGGATCAGCAAGCAGGAGTATG; β-actin reverse primer: 5’-GCATTTGCGGTGGACGAT. All amplifications were conducted in triplicate. All values were normalized to an endogenous β-actin control. The relative expression of mRNA was normalized with the control in each experiment.

**Western blot for phosphorylated AKT and MAPK**

Cells were seeded overnight and refreshed with serum-free medium, then subjected to serum starvation for 12 hours. After starvation, cells were cultured in the presence of PD98059 or LY294002, specific inhibitors of MAPK kinase and PI3K, respectively, for 1 hour prior to 10 nM IGF1 stimulation. Cells for detection of phosphorylated MAPK and AKT were collected after 10 minutes and 1 hour of IGF1 stimulation, respectively. Primary antibodies were used as follows: anti-AKT (#9272, Cell signaling), anti-phospho-AKT (#9271, Cell signaling), anti-phospho-MAPK (sc-7383, Santa Cruz), and anti-MAPK (sc-94, Santa Cruz).

**Cell cycle analysis**

Cells were fixed in 70% ethanol overnight and stained with propidium iodide (PI, Sigma). The DNA content were analyzed using flow cytometry (Calibur, BD, Franklin, NJ, USA ).

**Table S1** **Correlation between clinical parameters and relative expression of miR-99a in 40 oral squamous cell carcinoma (OSCC) patients^#^.**

| **Clinical features** | **Case number** | **Relative expression of miR-99a** | **p-value** |
| --- | --- | --- | --- |
| **Age**  <50  ≧50 | N=24  N=16 | 0.4550±0.04374  0.3695±0.04378 | 0.1928 |
| **TNM stage**  Stage I-II  Stage III-IV | N=15  N=25 | 0.4101±0.05014  0.4272±0.04194 | 0.7985 |
| **T classification**  T1-2  T3-4 | N=23  N=17 | 0.4061±0.03661  0.4407±0.05739 | 0.5981 |
| **N classification**  N=0  N=1-2 | N=23  N=17 | 0.4352±0.04199  0.4013±0.05009 | 0.6051 |
| **Lymphovascular invasion**  No  Yes | N=21  N=18 | 0.4931±0.04194  0.3358±0.04454 | 0.0144* |

#, all of these 40 patients were male patients without any evidence of distant metastasis disease prior to surgery (M0). None of them had N3 disease.

*, p<0.05 by student *t* test

**Table S2.** **Prediction of candidate targets of miR-99a by miRWalk and correlations between miR-99a expression and candidate gene expression in 40 pairs of oral squamous cell carcinoma (OSCC) (GSE37991) by Pearson correlation.**

#, 10 programs included DIANA-mT, miRanda, miRDB, miRwalk, RNAhybrid, PICTAR4, PICTAR5, PITA, RNA22, TargetScan.

| **Gene** | **miRWalk**  **(10 programs)^#^** | **Pearson correlation** | **p value** |
| --- | --- | --- | --- |
| **CDH2** | No | -0.1433 | 0.3778 |
| **VIM**  **TWIST1** | No  No | 0.05180  -0.01565 | 0.7509  0.9237 |
| **SNAI1** | No | 0.3706 | 0.0186* |
| **SNAI2** | No | -0.1340 | 0.4097 |
| **MMP2** | 1/10 | -0.02212 | 0.8922 |
| **MMP9** | No | -0.04309 | 0.7918 |
| **IGF1R** | 4/10 | -0.4055 | 0.0094 ** |
| **FRAP1 (mTOR)** | 10/10 | -0.2544 | 0.1132 |

No, Not predicted; * p<0.05 by Pearson correlation; ** p<0.01 by Pearson correlation

**Figure S1** **Over-expression of miR-99a did not change cell morphology.** (A) Ectopic miR-99a did not change cell morphology in miR-99a expressing OEC-M1 (OEC-M1 miR-99a) and CGHNC9 (CGHNC9 miR-99a) cells when compared with their non-silencing microRNA expressing controls, OEC-M1 NS and CGHNC9 NS under phase contrast microscopy with 400X magnification, respectively. (B) Immunofluorescence using anti-α-tubulin, (C) anti-E-cadherin and (D) anti-focal adherin kinase (FAK) showed similar patterns in OEC-M1 (OEC-M1 NS and OEC-M1 miR-99a) and CGHNC9 (CGHNC9 NS and CGHNC9 miR-99a) cells under fluorescent confocal microscope with 630X magnification (shown in grey mode).

**Figure S2 Expression of IGF1/IGFR1 in OSCC tissues and cells.** (A) The level of IGF1R mRNA was up-regulated in 22/40 (55%) of OSCC tissues with >2-fold increase by microarray analysis when compared with their corresponding nontumorous parts. Up-regulated IGF1 mRNA was not detectable in 40 pairs if OSCC tissues. (B) Immunoblot assay for detection of IGF1R protein in two independent batches of HOK and OSCC cells (upper panel). The protein levels were normalized against an internal control β-actin. Ratios were determined by dividing the normalized protein levels in OSCC cells with that in HOK cells. The mean of ratio in the graphs was measured by averaging the ratios from two independent blots (lower panel). Bar, SE.

**Figure S3 Qunatification of IGF1R and mTOR mRNA in miR-99a expressing OSCC cells.** Quantitative RT-PCR demonstrated the relative mRNA levels for IGF1R and mTOR in OEC-M1 and SCC15 cells with ectopic miR-99a expression (OEC-M1 miR-99a and SCC15 miR-99a) or non-silencing microRNA expressing controls (OEC-M1 NS and SCC15 NS). All amplifications were normalized to an endogenous β-actin control. The relative expression of mRNA in miR-99a expressing cells was normalized to that in non-silencing microRNA expressing controls. Bar, SE; ***, p<0.001.

**Figure S4** **IGF1R rescued the inhibition of migration and invasion in miR-99a expressing OEC-M1 cells.** (A) Protein levels of IGF1R expression were determined by Western blot in miR-99a expressing OEC-M1 (OEC-M1 miR-99a) and non-silencing microRNA expressing controls (OEC-M1 NS) with ectopic IGF1R expression. α-tubulin served as a loading control. (B) Representative data showed the relative migration/invasion activity of OEC-M1 NS and OEC-M1 miR-99a cells expressing IGF1R (OEC-M1 NS/IGF1R and OEC-M1 miR-99a/IGF1R) and their vector controls (OEC-M1 NS/VC and OEC-M1 miR-99a/VC). The relative migration/invasion activity was defined by normalizing the mean of migrated or invaded cells/per field in cells expressing IGF1R to that in OEC-M1 NS/VC. Bar, SE; * p<0.1; *** p<0.001. (C) Levels of miR-99a were determined by qRT-PCR in OEC-M1 NS cells with ectopic IGF1R expression. MiR-99a expression was normalized against an endogenous control U6. The relative expression of miR-99a was presented by normalizing miR-99a expression in OEC-M1 NS cells with ectopic IGF1R expression (OEC-M1 NS/IGF1R) to that in the controls (OEC-M1 NS/VC). Bar, SE; *** p<0.001.

**Figure S5 Activation of AKT and MAPK by IGF1 stimulation was inhibited upon treatment with the PI3K inhibitor LY294002 and MAPK kinase inhibitor PD98059, respectively.** After serum starvation, cells were treated with vehicle, 10 nM IGF1, or combination of LY294002/PD98059 and IGF1. Immunoblot assay showed that levels of phosphorylated AKT and MAPK in IGF1-stimulated OEC-M1 cells were inhibited upon treatment with LY294002 and PD98059, respectively.

**Figure S6 Ectopic miR-99a expression did not change cell cycle but subtly affected the expression of cell cycle-related proteins.** (A) Ectopic miR-99a expression did not change the cell cycle in OEC-M1 and CGHNC9 cells using propidium iodide staining. (B) Immunoblot analysis of cell cycle-related molecules, including cyclin D, cyclin E, p21 and p27 in OEC-M1 and CGHNC9 cells with ectopic miR-99a expression (OEC-M1 miR-99a and CGHNC9 miR-99a) or non-silencing microRNA expressing controls (OEC-M1 NS and CGHNC9 NS). α-tubulin served as an internal control.
