# Supplementary figures and images for: Reciprocal regulation of MicroRNA-99a and insulin-like growth factor I receptor signaling in oral squamous cell carcinoma cells
Source: Mol Cancer. 2014 Jan 10;13:6. doi: 10.1186/1476-4598-13-6 (PMC3895693; doi:10.1186/1476-4598-13-6)

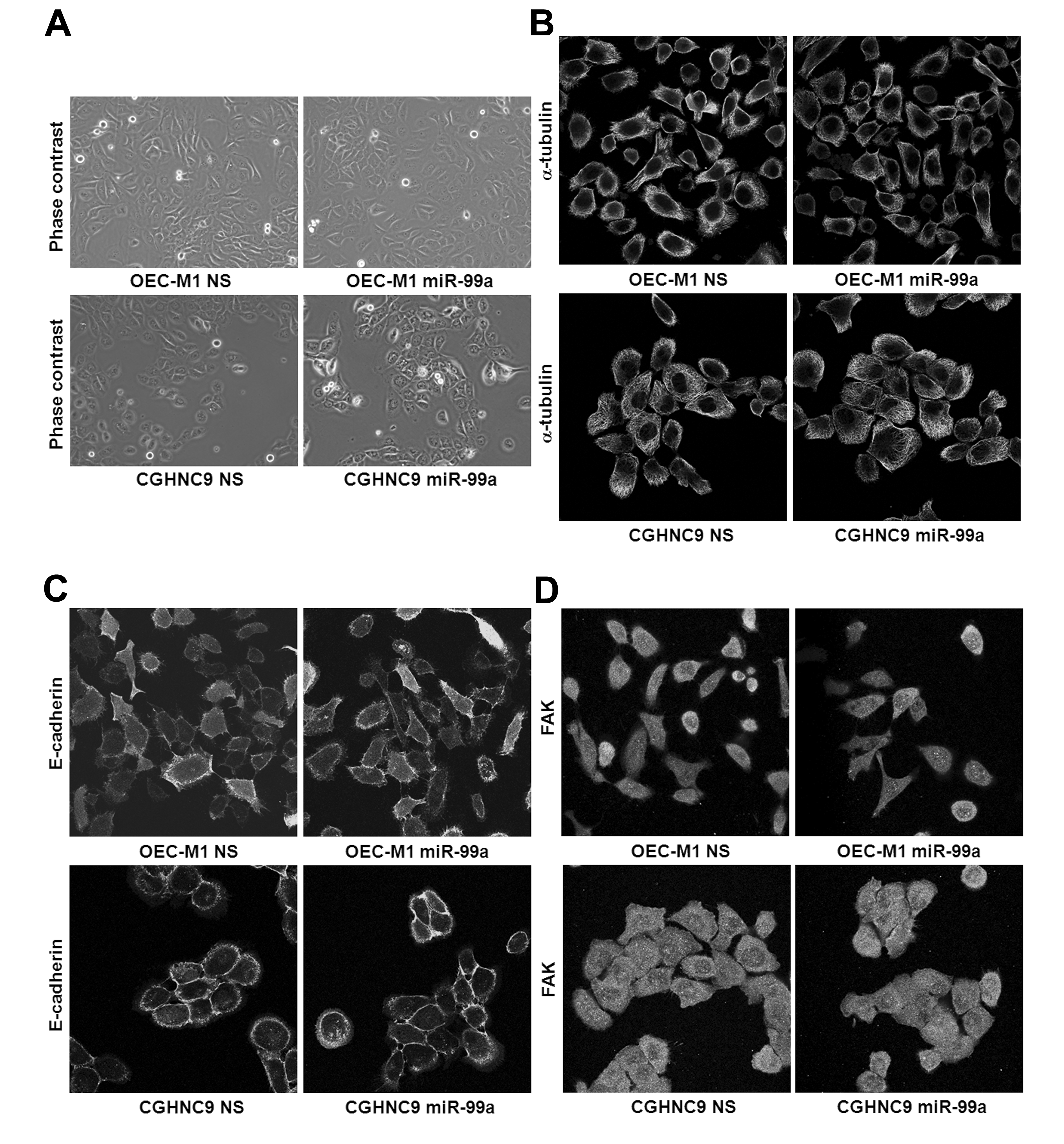

Supplement: Additional file 2: Figure S1 — Over-expression of miR-99a did not change cell morphology. (A) Ectopic miR-99a did not change cell morphology in miR-99a expressing OEC-M1 (OEC-M1 miR-99a) and CGHNC9 (CGHNC9 miR-99a) cells when compared with their non-silencing microRNA expressing controls, OEC-M1 NS and CGHNC9 NS under phase contrast microscopy with 400X magnification, respectively. (B) Immunofluorescence using anti-α-tubulin, (C) anti-E-cadherin and (D) anti-focal adherin kinase (FAK) showed similar patterns in OEC-M1 (OEC-M1 NS and OEC-M1 miR-99a) and CGHNC9 (CGHNC9 NS and CGHNC9 miR-99a) cells under fluorescent confocal microscope with 630X magnification (shown in grey mode). [file 1476-4598-13-6-S2.tiff]

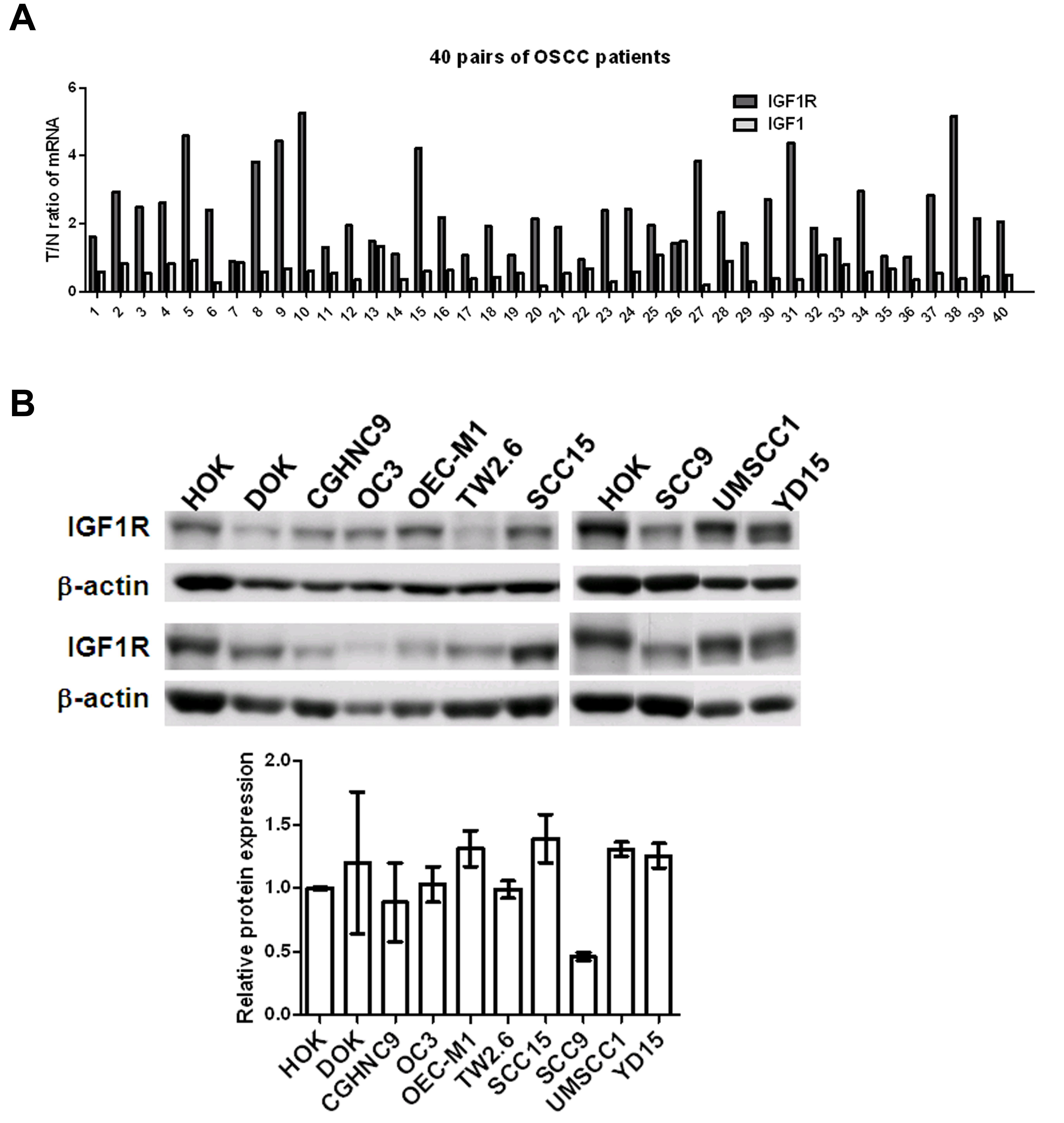

Supplement: Additional file 3: Figure S2 — Expression of IGF1/IGFR1 in OSCC tissues and cells. (A) The level of IGF1R mRNA was up-regulated in 22/40 (55%) of OSCC tissues with >2-fold increase by microarray analysis when compared with their corresponding nontumorous parts. Up-regulated IGF1 mRNA was not detectable in 40 pairs of OSCC tissues. (B) Immunoblot assay for detection of IGF1R protein in two independent batches of HOK and OSCC cells (upper panel). The protein levels were normalized against an internal control β-actin. Ratios were determined by dividing the normalized protein levels in OSCC cells with that in HOK cells. The mean of ratio in the graphs was measured by averaging the ratios from two independent blots (lower panel). Bar, SE. [file 1476-4598-13-6-S3.tiff]

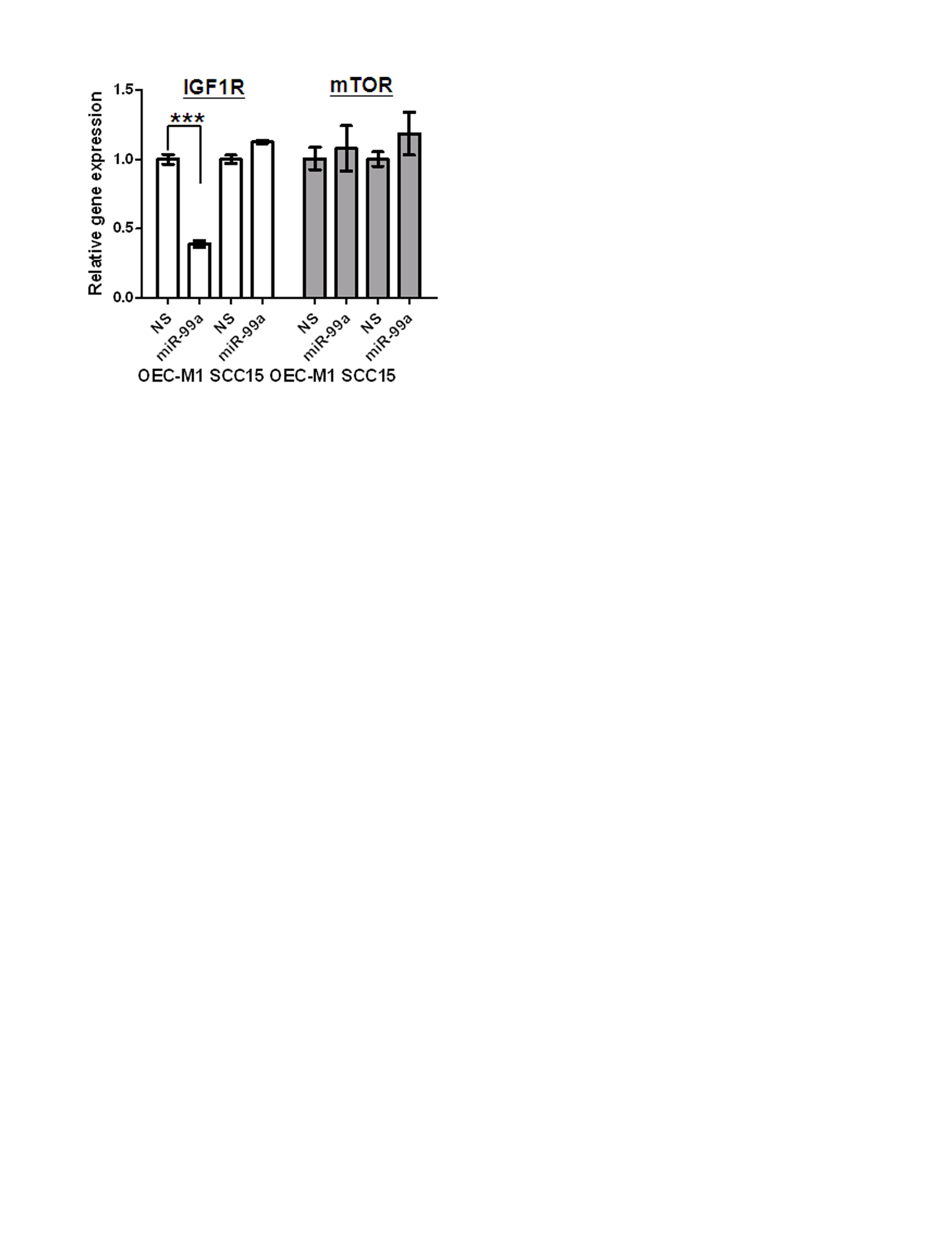

Supplement: Additional file 4: Figure S3 — Qunatification of IGF1R and mTOR mRNA in miR-99a expressing OSCC cells. Quantitative RT-PCR demonstrated the relative mRNA levels for IGF1R and mTOR in OEC-M1 and SCC15 cells with ectopic miR-99a expression (OEC-M1 miR-99a and SCC15 miR-99a) or non-silencing microRNA expressing controls (OEC-M1 NS and SCC15 NS). All amplifications were normalized to an endogenous β-actin control. The relative expression of mRNA in miR-99a expressing cells was normalized to that in non-silencing microRNA expressing controls. Bar, SE; ***, p < 0.001. [file 1476-4598-13-6-S4.tiff]

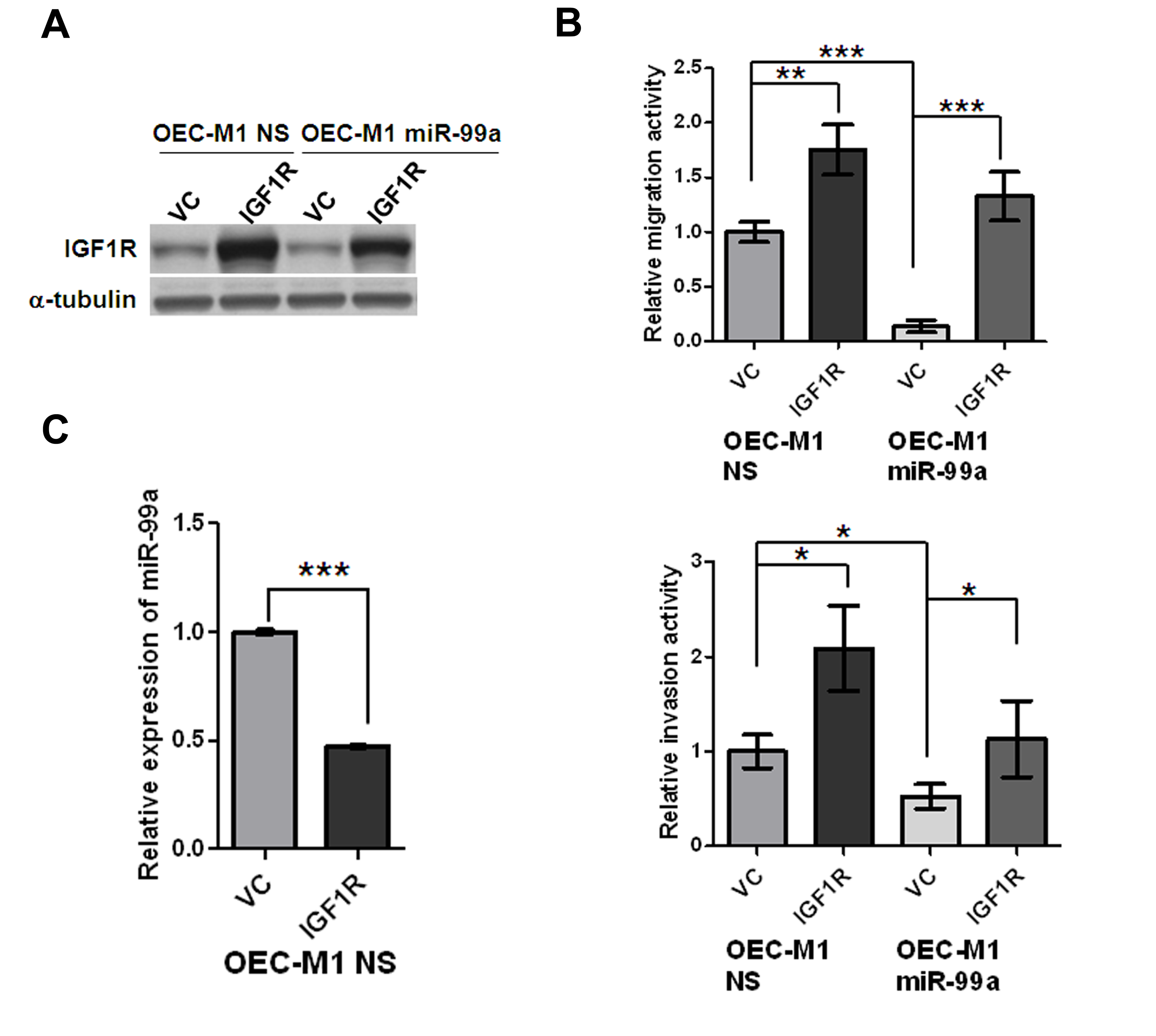

Supplement: Additional file 5: Figure S4 — Figure S4 IGF1R rescued the inhibition of migration and invasion in miR-99a expressing OEC-M1 cells. (A) Protein levels of IGF1R expression were determined by Western blot in miR-99a expressing OEC-M1 (OEC-M1 miR-99a) cells and non-silencing microRNA expressing controls (OEC-M1 NS) with ectopic IGF1R expression. α-tubulin served as a loading control. (B) Representative data showed the relative migration/invasion activity of OEC-M1 NS and OEC-M1 miR-99a cells expressing IGF1R (OEC-M1 NS/IGF1R and OEC-M1 miR-99a/IGF1R) and their vector controls (OEC-M1 NS/VC and OEC-M1 miR-99a/VC). The relative migration/invasion activity was defined by normalizing the mean of migrated or invaded cells/per field in cells expressing IGF1R to that in OEC-M1 NS/VC. Bar, SE; *p < 0.1; ***p < 0.001. (C) Levels of miR-99a were determined by qRT-PCR in OEC-M1 NS cells with ectopic IGF1R expression. MiR-99a expression was normalized against an endogenous control U6. The relative expression of miR-99a was presented by normalizing miR-99a expression in OEC-M1 NS cells with ectopic IGF1R expression (OEC-M1 NS/IGF1R) to that in the controls (OEC-M1 NS/VC). Bar, SE; *** p < 0.001. [file 1476-4598-13-6-S5.tiff]

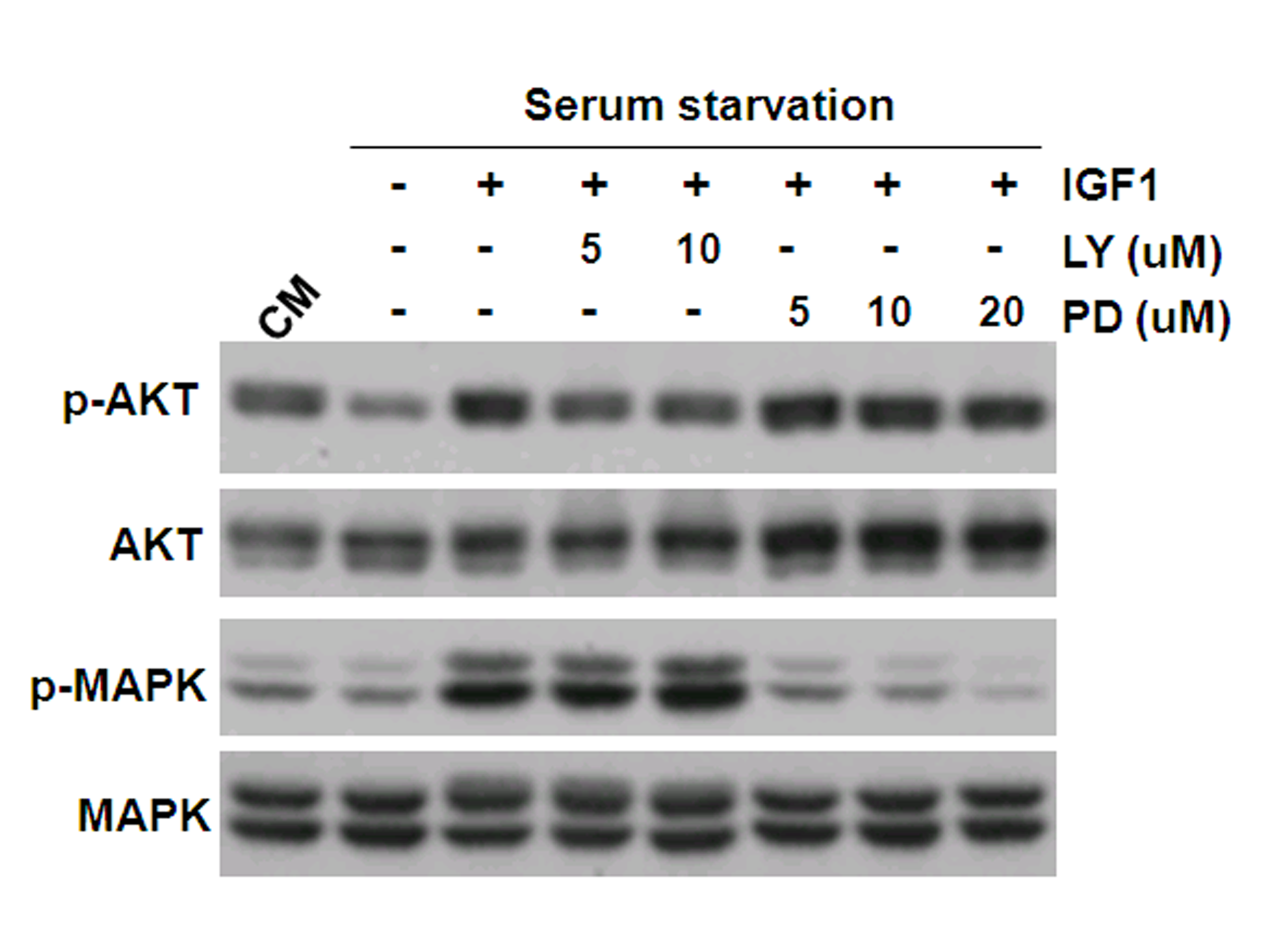

Supplement: Additional file 6: Figure S5 — Activation of AKT and MAPK by IGF1 stimulation was inhibited upon treatment with the PI3K inhibitor LY294002 and MAPK kinase inhibitor PD98059, respectively. After serum starvation, cells were treated with vehicle, 10 nM IGF1, or combination of LY294002/PD98059 and IGF1. Immunoblot assay showed that levels of phosphorylated AKT and MAPK in IGF1-stimulated OEC-M1 cells were inhibited upon treatment with LY294002 and PD98059, respectively. [file 1476-4598-13-6-S6.tiff]

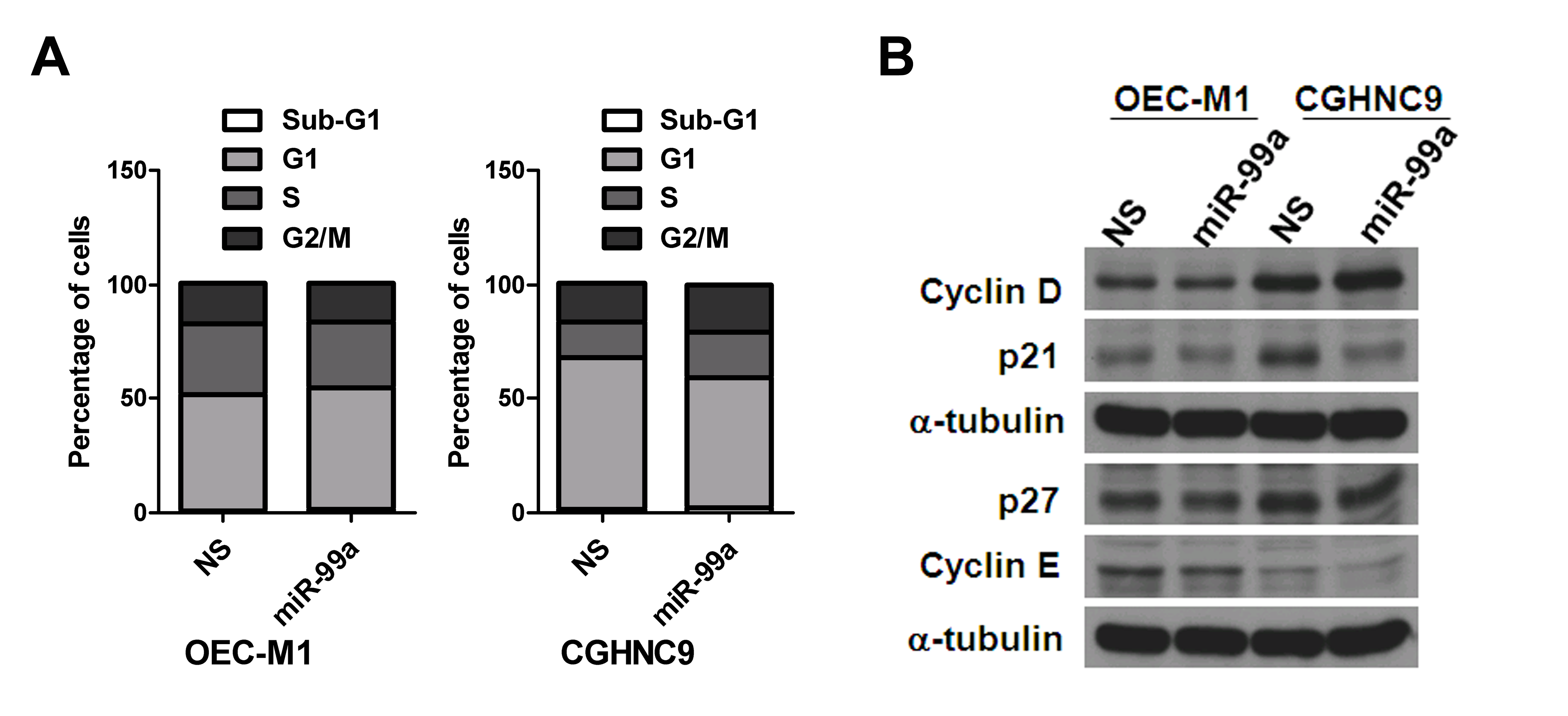

Supplement: Additional file 7: Figure S6 — Ectopic miR-99a expression did not change cell cycle but subtly affected the expression of cell cycle-related proteins. (A) Ectopic miR-99a expression did not change the cell cycle in OEC-M1 and CGHNC9 cells using propidium iodide staining. (B) Immunoblot analysis of cell cycle-related molecules, including cyclin D, cyclin E, p21 and p27 in OEC-M1 and CGHNC9 cells with ectopic miR-99a expression (OEC-M1 miR-99a and CGHNC9 miR-99a) or non-silencing microRNA expressing controls (OEC-M1 NS and CGHNC9 NS). α-tubulin served as an internal control. [file 1476-4598-13-6-S7.tiff]
